# Global Protective Ventilator Settings Are Not Regional Protection: A Compartment-Resolved Framework for Personalizing Mechanical Ventilation in Acute Respiratory Distress Syndrome

**DOI:** 10.3390/jcm15197545

**Published:** 2026-09-28

**Authors:** Michael S. Champagne

**Affiliations:** VentGuard Analytics, LLC, Webster, TX 77598, USA; mchampagne@ventguard.ai

**Keywords:** acute respiratory distress syndrome, ventilator-induced lung injury, lung-protective ventilation, regional lung mechanics, repeated alveolar collapse and expansion, positive end-expiratory pressure, tidal volume, in silico modeling, clinical decision support

## Abstract

**Background**: Lung-protective ventilation in acute respiratory distress syndrome (ARDS) is guided by whole-lung measures, although the same acceptable breath may stabilize regions undergoing repeated alveolar collapse and expansion (RACE) while overdistending tissue already open. Fatigue-Aware Alveolar Stress and Strain Tracking (FAAST) is a clinician-in-the-loop framework designed to make this regional tradeoff observable. **Methods**: We first constructed a deterministic, twenty-compartment, phenotype-conditioned model from published observations of acute lung injury. With FAAST excluded, the test lung was evaluated controller-blind against nine prespecified correspondence targets under conventional ventilation and frozen after all nine were met. FAAST was then applied in four experiments examining alternative ventilator strategies, regional observability, a recruitment–overdistension crossover, and the interaction between recruitment and tidal-volume reduction. **Results**: Before FAAST analysis, the frozen phenotype model met all nine prespecified correspondence targets. FAAST analysis then showed that globally acceptable pressures could coexist with suprathreshold regional loading, while a summed-dose controller obscured opposing regional states. At 96 h, median modeled structural-compliance loss was 61.4% at positive end-expiratory pressure (PEEP) 8 cm H_2_O, 40.7% with the FAAST crossover at mean PEEP 13.5 cm H_2_O, and 39.3% at fixed PEEP 15. In a fresh virtual cohort, the crossover remained favorable to PEEP 8 in every matched case. Tidal-volume reduction lowered loss by 1.2 points at practice PEEP and 5.9 points after recruitment, with a 4.4-point interaction. No strategy eliminated regional injury. **Conclusions**: Within this model, global protection did not ensure regional protection. Compartmental analysis preserved information lost through aggregation and generated hypotheses for matching the complete ventilator pattern to lung state. These model-conditional findings do not establish clinical efficacy or a validated PEEP target; external waveform, imaging, physiologic, and outcome validation is required before clinical use.

## 1. Introduction

The ARDS Network trial changed mechanical ventilation by demonstrating that lower tidal-volume ventilation reduces mortality in patients with acute respiratory distress syndrome (ARDS). Its central implication was not simply that smaller breaths are safer, but that the mechanical burden imposed by ventilation can alter the course of lung injury and survival [1]. That distinction remains central. Mechanical ventilation is not simply supportive; in the wrong mechanical environment, it becomes part of the injury process.

That achievement was durable, but it did not solve the larger problem. A secondary analysis of ARDSNet trials reported a decline in ARDS mortality from 35.4% in 1996 to 28.3% in 2013, plausibly reflecting advances in fluid management, tidal-volume reduction, limitation of plateau pressure, and the application of PEEP [2,3]. More recent analyses have not demonstrated a continuing downward trend, and contemporary international cohorts still report substantial mortality across ARDS severity strata [4,5]. The field made a landmark advance, but the mortality curve has proved difficult to bend further. We still lack a sufficiently resolved mechanical account of why some patients stabilize while others deteriorate despite meeting conventional lung-protective targets.

The years since ARDSNet have therefore been a sustained effort to characterize ventilator load more faithfully. Plateau pressure provided a safety ceiling for end-inspiratory distending pressure. Driving pressure related tidal volume to the compliance of the respiratory system available to receive it [6]. Mechanical power assembled pressure, volume, flow, and respiratory rate into a more complete energetic description of ventilation [7,8,9]. Each was a genuine advance, and each added important information to the clinical assessment of the ventilated lung. Yet none fundamentally changed the resolution at which that lung is observed.

At the airway, a single pressure–volume waveform represents the summed behavior of regions that may differ widely in aeration, compliance, resistance, recruitability, and prior injury. It can describe the response of the respiratory system as a whole, but not which regions accepted the breath, remained closed, underwent repeated alveolar collapse and expansion, or exceeded their mechanical tolerance. We have become increasingly sophisticated in describing the global ventilator–lung interaction without acquiring comparable intelligence about its regional consequences. The lung does not experience a ventilator setting in isolation; it experiences the regional stress, deformation, instability, and recovery produced when the complete pattern of ventilation is applied to its current mechanical state. What remains unavailable at the bedside is the regional mechanical intelligence needed to interpret the settings already in use.

PEEP provides the clearest clinical illustration of this limitation, although it is only one component of the ventilator prescription. Its importance lies partly in establishing the end-expiratory state and determining how much lung remains available to receive the next tidal breath. In a recruitable region, sufficient end-expiratory pressure may stabilize an unstable unit and reduce repeated alveolar collapse and expansion (RACE). In tissue already open, the same pressure may increase static deformation and promote overdistension. Schiller and colleagues directly visualized RACE in a surfactant-depletion model and demonstrated that PEEP could stabilize alveoli across a range of tidal volumes [10]. Gaver and colleagues later showed that recruitment–derecruitment accounted for only 2–5% of total dissipated energy in a porcine ARDS model, yet concentrated substantial power over a small area and brief event duration [9]. The relevant question is therefore not whether PEEP should universally be higher or lower, but when the benefit of stabilizing recruitable lung is offset by increasing deformation elsewhere. This regional opposition offers a plausible explanation for the mixed results of population-level higher-PEEP strategies [11,12].

The problem arises because the ventilated lung is not a single mechanical compartment, and in ARDS that limitation becomes extreme. Computed tomography established the “baby lung” concept: the aerated and ventilatable portion may represent only a fraction of the anatomical lung, and the prescribed tidal volume must be accommodated by whatever tissue remains open [13]. Superimposed pressure, surfactant dysfunction, edema, consolidation, and mechanical interdependence further create regional differences in opening pressure, recruitability, and local stress concentration [14,15]. A tidal volume of 6 mL/kg predicted body weight and a plateau pressure below 30 cm H_2_O therefore cannot ensure that every region is being ventilated protectively. Terragni and colleagues demonstrated this clinically when computed tomography identified tidal hyperinflation in approximately one third of patients ventilated within those limits, accompanied by higher inflammatory mediator concentrations [16]. The limitation is not the principle of lung-protective ventilation, but the spatial resolution at which protection is assessed.

Panwar and colleagues’ analysis of 1117 patients enrolled in LUNG SAFE provides a clinically grounded way to characterize the lung state to which ventilation is applied [17]. Respiratory-system compliance ranged widely: 12% of patients had preserved compliance, 14% occupied an intermediate range, and 74% had poor compliance. Compliance and oxygenation were also nearly dissociated, demonstrating that neither variable alone adequately describes the injured lung. These phenotypes do not provide a measured regional map, but they offer an observed clinical framework from which a reduced-order lung representation can be constructed. In the present study, the Panwar strata define the phenotype-level starting state, while a twenty-compartment representation adds regional differences in mechanical participation, opening pressure, recruitability, and susceptibility. The phenotype model describes the lung to which ventilation is applied; FAAST examines how the complete ventilator pattern is distributed within it.

We therefore introduce Fatigue-Aware Alveolar Stress and Strain Tracking (FAAST) as a clinician-in-the-loop analytical framework intended to address this observability gap. FAAST extends lung-protective ventilation rather than replacing it, carrying the ARDSNet principle from limiting whole-lung mechanical burden toward estimating its regional distribution. The controls remain familiar and global, but their effects are interpreted together through a phenotype-resolved representation of the injured lung. PEEP helps determine how much lung is recruited and remains available; tidal volume determines the deformation imposed on that participating lung; and respiratory rate, inspiratory time, and flow determine how frequently and in what manner the exposure is delivered. FAAST maps this complete pattern into estimates of regional participation, RACE, overdistension, stress, strain, cumulative mechanical exposure, and evolving modeled damage (Figure 1). In practical terms, it examines the mechanical economy of the breath: how much lung can be made stably available for ventilation, and at what regional mechanical cost. PEEP selection is therefore an important application of FAAST, but not its defining purpose.

The present study brings together two related elements: a reduced-order, twenty-compartment lung model representing clinically recognizable differences in regional mechanics, and the FAAST theoretical engine described in the companion paper [18]. The engine translates ventilator inputs and regional responses into compartment-level measures of mechanical exposure and modeled injury state. The lung model was correspondence-tested against external conventional-ventilation targets with FAAST excluded, then frozen before the proposed strategy was evaluated. This controller-blind sequence prevents the lung representation from being adjusted simply to favor FAAST. The framework does not reproduce an individual patient or claim alveolar-scale precision. It provides a transparent environment in which familiar ventilator decisions can be examined at a regional resolution unavailable from routine global measurements.

FAAST is not presented as an autonomous controller or a substitute for clinical judgment. It supplies a model-estimated regional mechanical signal that remains subordinate to oxygenation, ventilation, acid–base status, hemodynamics, right-ventricular tolerance, and the clinician’s assessment. Its translational proposition is narrower and testable: regional mechanical information may reveal RACE–overdistension tradeoffs and cumulative exposure patterns that conventional whole-lung variables cannot resolve. Any adjustment remains a global ventilator intervention applied by the clinician, followed by reassessment of mechanical response and physiologic tolerance.

To examine this proposition, we performed a deterministic in silico study using the frozen twenty-compartment lung representation. Four experiments assessed conventional injury correspondence, regional burden hidden by global measurements, the model-based crossover between residual RACE and overdistension during PEEP titration, and the interaction between recruitment and tidal-volume reduction. We hypothesized that globally protective settings would not distribute mechanical burden uniformly and that compartment-resolved analysis would reveal tradeoffs concealed by whole-lung summaries. The resulting conclusions are conditional on the specified model and are offered as falsifiable mechanical predictions requiring external validation, not as evidence of clinical efficacy.

## 2. Methods

### 2.1. Study Design and Developmental Sequence

We performed a deterministic in silico developmental study to examine what Fatigue-Aware Alveolar Stress and Strain Tracking (FAAST) would reveal when complete ventilator prescriptions were applied to lungs with different mechanical states. The purpose was not to reconstruct individual patients or establish clinical efficacy. We asked whether compartment-resolved analysis could preserve regional mechanical information lost through whole-lung aggregation and generate physiologically coherent, testable hypotheses for ventilator management.

The study brought together two distinct but computationally linked components. The first was the FAAST analytical engine, which translates the applied ventilator pattern into regional estimates of mechanical participation, repeated alveolar collapse and expansion (RACE), overdistension, stress, strain, cumulative exposure, and modeled tissue state [18]. The second was a reduced-order lung model comprising twenty mechanically heterogeneous compartments. This model supplied the phenotype-conditioned lung state on which FAAST operated. In practical terms, the substrate defined the lung receiving the breath, while FAAST estimated how that breath was distributed and what regional mechanical consequences followed.

Model construction and intervention testing were separated to limit the risk of tuning the substrate until a preferred FAAST strategy succeeded. The phenotype-conditioned lung model was first evaluated under conventional ventilation against nine prespecified correspondence targets in 200 virtual lungs generated with seed 20260710. FAAST-guided management was excluded during this phase, and no FAAST outcome was used to adjust phenotype distributions, recruitability, regional mechanics, injury kinetics, or correspondence tolerances. Once all nine targets were satisfied, the model and its parameters were frozen before strategy testing; the complete controller-blind correspondence fence is provided in Table S1.

Four experiments were then performed using the frozen substrate. They compared ventilation strategies, examined regional injury hidden by globally acceptable measurements, evaluated a recruitment–overdistension crossover, and tested whether the modeled effect of tidal-volume reduction depended on the preceding recruitment state. Where strategies were compared, the same virtual lung was evaluated under each intervention so that differences arose from the applied ventilator strategy rather than from differences in the starting lung. Table 1 summarizes the experimental sequence. Complete equations, parameter definitions, correspondence criteria, and sensitivity analyses are provided in the Supplementary Methods. The accompanying frozen Zenodo release [19] archives the investigator-developed FAAST-2 Regional Lung Intelligence implementation (version 1.0.0), together with the random seeds and execution instructions. The implementation was executed in Python version 3.12.3 (Python Software Foundation, Wilmington, DE, USA) using NumPy version 2.4.4 (NumPy Developers; NumFOCUS, Austin, TX, USA), with no other analytical software dependencies.

An expanded experimental roadmap and the matched 2 × 2 factorial structure for Experiment 4 are provided in Tables S2 and S3, respectively.

### 2.2. FAAST Analytical Engine and Phenotype-Conditioned Test Model

FAAST is a clinician-in-the-loop analytical engine for examining the mechanical economy of the complete breath. It evaluates tidal volume, positive end-expiratory pressure (PEEP), respiratory rate, inspiratory time, and the applied pressure–flow pattern as interacting boundary conditions rather than as isolated settings. These inputs are applied to a defined regional lung state. FAAST then estimates which compartments participate in ventilation, how tidal volume is distributed among them, and whether individual regions remain stable, undergo repeated alveolar collapse and expansion (RACE), or enter an overdistended state. Its purpose in this study was therefore broader than PEEP selection: it was to examine the regional consequences of applying a complete ventilator prescription to a lung with finite and uneven mechanical reserve.

RACE was represented as recruitment and derecruitment of a compartment between its end-expiratory and end-inspiratory states. This formulation reflects the repeated alveolar collapse and expansion described experimentally in acute lung injury [10,20]. Increasing PEEP may stabilize recruitable compartments and reduce RACE, but the newly available lung does not receive volume in isolation. Recruitment changes the distribution of the entire tidal breath and may transfer deformation toward compartments that are already open. FAAST was designed to preserve this regional tradeoff rather than collapse recruitment and overdistension into a single whole-lung measurement.

The engine separates regional mechanical exposure from its modeled tissue consequence. Stress and strain are calculated for each participating compartment and accumulated as Time-Accumulated Stress History (TASH) and Strain-Accumulated Strain History (SASH). The Continuum of Damage Index (CDI) retains the evolving tissue state produced by that exposure, including the effects of repeated instability, barrier integrity, recovery, and structural memory. This distinction is important because a decline in subsequent mechanical exposure does not necessarily indicate recovery. It may reflect stabilization of the compartment, but it may also occur after the compartment has stiffened and accepts less of the delivered breath. Full definitions and equations for these variables are provided in the Supplementary Methods.

A defined lung state was required to test the FAAST engine. We therefore constructed a reduced-order model containing twenty parallel compartments distributed along a gravitational axis. These compartments represent mechanically distinguishable regions rather than individual anatomical alveoli. They differ in structural compliance, superimposed pressure, opening pressure, recruitability, surfactant state, epithelial-barrier integrity, and antecedent injury. Twenty compartments provided sufficient resolution to represent graded regional heterogeneity while preserving an interpretable and reproducible model structure.

The phenotype distribution and its global mechanical starting conditions were anchored to Panwar and colleagues’ analysis of 1117 patients enrolled in LUNG SAFE [17]. Their compliance phenotypes supplied a clinically observed framework for representing preserved, intermediate, and progressively reduced mechanical reserve in ARDS. The poor-compliance population was further divided into graded severity levels so that progressive loss of available lung could be examined without treating all low-compliance lungs as mechanically equivalent. The resulting phenotype assignments were population-level approximations derived from published summary data; they were not reconstructions of individual LUNG SAFE patients.

Park and colleagues’ observations in patients receiving long-term ventilation for amyotrophic lateral sclerosis were used separately as a chronic-stability comparator [21]. These patients were not treated as an ARDS phenotype, and their outcomes were not used to validate CDI or exclude microscopic injury. Instead, their reported ventilator conditions provided a homeostatic floor: a preserved-mechanics reference condition under which prolonged ventilation should not inevitably produce progressive modeled failure. This boundary was included to ensure that the engine could represent mechanical stability as well as injury progression.

The two components remained distinct throughout the study. The phenotype model defined the regional lung condition receiving ventilation; FAAST analyzed how the prescribed breath was distributed within that condition and how regional exposure accumulated over time. This separation allowed the lung representation to be correspondence-tested and frozen before FAAST-guided strategies were introduced. The detailed compartment equations, parameter assignments, state-update rules, and sensitivity analyses are reported in the Supplementary Methods.

### 2.3. Controller-Blind Correspondence Testing and Model Freeze

A principal methodological risk in a model of this kind is that its assumptions or free parameters might be adjusted after the performance of an intervention is known. We addressed this risk by separating construction of the phenotype-conditioned lung model from subsequent testing of FAAST. During the correspondence phase, the model was evaluated only under conventional ventilation. FAAST-guided strategies were excluded, and no FAAST result was used to determine the phenotype distribution, recruitability, baseline mechanics, injury kinetics, or correspondence tolerances. The intervention experiments began only after the prespecified correspondence fence had been satisfied.

We use correspondence deliberately rather than validation. No patient-level dataset available to this study contained synchronized ventilator waveforms, regional recruitment, RACE activity, local deformation, and longitudinal tissue-injury measurements. The purpose of this phase was therefore narrower: to determine whether the reduced-order model occupied a clinically recognizable mechanical domain and reproduced the expected direction and temporal behavior of conventionally ventilated acute lung injury. Passing the correspondence fence indicates that the model was not materially inconsistent with the selected observations. It does not establish patient-specific predictive accuracy or clinical effectiveness.

The fence contained nine prespecified pass-or-fail gates evaluated in 200 virtual lungs generated with seed 20260710 (Table 2). Seven gates examined phenotype distribution, recruitability, baseline mechanics, injury onset and trajectory, severity ordering, and regional stress amplification. Two additional gates enforced clinically or experimentally anchored protocol boundaries: the plateau-pressure range associated with tidal-volume reduction and the maximum permitted rate of structural-compliance decline. All nine gates were satisfied before FAAST-guided strategies were tested.

Two changes were made during model construction to improve correspondence with the external anchors. Recruitability was changed from a symmetric to a right-skewed distribution to reproduce the reported relationship between its mean and median. An upper boundary was also placed on the rate of structural-compliance decline using the steepest rate reported in the selected large-animal acute-lung-injury study. Both changes preceded FAAST testing and were made without reference to intervention performance.

A subsequent code review identified a pathway-gating error in which epithelial-barrier state had been applied to both modeled injury pathways. The logic was corrected so that barrier failure modifies RACE-mediated atelectrauma, whereas overdistension can injure an otherwise intact compartment. The complete correspondence fence and all four experiments were then rerun using the corrected implementation. No parameter was retuned to preserve a preferred strategy result, and the corrected implementation is the frozen version reported here and deposited with the reproducibility package [19].

### 2.4. Compartmental Lung Architecture and Per-Breath Mechanics

Each virtual lung contained twenty parallel respiratory compartments arranged from nondependent to dependent along a gravitational axis. The compartments shared the applied airway pressure but differed in structural compliance, superimposed pressure, opening pressure, recruitability, surfactant state, epithelial-barrier integrity, and prior modeled injury. They represent mechanically distinguishable regions rather than individual anatomical alveoli. This architecture increased the model’s resolution from one whole-lung value to twenty state-bearing elements that could respond differently to the same ventilator breath.

Antecedent ARDS severity was encoded in the initial lung architecture. Increasing severity reduced functional residual capacity, increased gravitational and opening-pressure gradients, lowered initial barrier integrity in dependent regions, and increased the proportion of poorly recruitable tissue. Native lung injury therefore entered the model as the condition present when ventilation began. The model did not impose a separate time-varying inflammatory process, because sufficient data were not available to define those kinetics responsibly. This boundary allowed the experiments to isolate how ventilation interacted with an already injured and mechanically heterogeneous lung.

At each representative breath, compartmental openness was calculated at end expiration and end inspiration from the pressure available to the compartment relative to its opening pressure. The prescribed tidal volume was then distributed according to each compartment’s openness and structural compliance. Closed or refractory compartments accepted little of the breath, while compartments that remained open and compliant accepted a larger share. This load-shifting mechanism allowed the same whole-lung tidal volume to produce different regional deformation as the amount and distribution of mechanically available lung changed.

Repeated alveolar collapse and expansion was represented by the change in compartmental openness across the breath, weighted by residual closure at end expiration. A compartment that remained open throughout the respiratory cycle had little or no RACE activity. A compartment that opened during inspiration and returned toward closure during expiration produced a larger RACE signal. This is a reduced-order representation of the experimentally described process rather than direct visualization of individual alveoli [10,20]. It allowed regional instability to be followed across breaths and compared with the opposing development of overdistension as PEEP increased.

Regional strain was calculated from local volume change using the specific-elastance relationship, expressed as linear deformation through a cube-root transformation [25]. Effective regional stress incorporated deformation-related stress, concentration of stress at interfaces between mechanically dissimilar compartments, and a rate-dependent viscoelastic contribution [15,26,27]. The suprathreshold stress ratio, $r_{\sigma }$, expressed effective stress relative to the model’s reference stress scale; $r_{\sigma }>1$ identified a compartment that had entered the model-defined suprathreshold loading regime. Neither the strain nor stress boundary should be interpreted as a universal alveolar rupture threshold or a diagnosis of histological injury.

The complete openness, volume-allocation, RACE, strain, and stress equations are provided in Supplementary Section S2, and the compartmental mechanics and threshold parameters are listed in Table S4, with parameter values and provenance in Supplementary Section S5. The deposited experimental implementation calculates linear strain using the cube-root specific-elastance formulation, whereas the companion theoretical framework uses a volumetric strain convention [18]. All thresholds, ratios, and experimental outcomes reported in this study refer to the deposited implementation.

### 2.5. FAAST Regional Dose and Tissue-State Architecture

The per-breath calculations determine where each breath is delivered and what regional stress, strain, and RACE activity it produces. FAAST adds a longitudinal layer by tracking how these exposures accumulate and how the modeled tissue state changes over time. The framework therefore distinguishes the ventilator prescription, the mechanical state of the lung, the regional burden delivered by each breath, and the tissue response assigned to that burden. These quantities are related, but they are not interchangeable.

The general FAAST framework is intended to analyze the complete ventilator prescription. In the present prototype, PEEP, tidal volume, respiratory rate, and inspiratory time entered the mechanical calculations. PEEP and tidal volume were varied in the intervention experiments, whereas respiratory rate and inspiratory time were held at their stated protocol values. Detailed inspiratory-flow and waveform behavior was not independently simulated in this build. The experiments should therefore be interpreted as a reduced-order evaluation of regional dose allocation and management logic rather than a complete representation of ventilator and respiratory-system dynamics.

TASH and SASH are not summed and then inserted into the Continuum of Damage Index (CDI). They describe the burden delivered to each compartment. CDI is advanced through a separate normalized and threshold-sensitive injury calculation, avoiding repeated attribution of the same exposure merely because it is also reported as TASH or SASH. This distinction allows the model to represent a stiff compartment that carries substantial stress with limited deformation differently from a compliant or unstable compartment that absorbs greater cyclic deformation under the same airway pressure.

The model separates two injury pathways because they do not depend on tissue state in the same way. Suprathreshold strain and stress act directly; an overdistended compartment does not require prior epithelial-barrier failure to sustain modeled injury from stretch. RACE enters through a separate atelectrauma pathway whose structural consequence increases as epithelial-barrier integrity declines. The model therefore permits a limited period of tolerance to repeated instability, followed by accelerated deterioration after the barrier state crosses its modeled resilience region. This lag followed by more rapid injury is consistent with experimental observations of epithelial-barrier response to repeated atelectrauma [24], but it is not intended as a complete model of cellular injury or inflammation.

CDI is a bounded state variable ranging from 0 to 1. It incorporates the competing effects of modeled injury, recovery, and retained structural memory and is linked by a predefined equation to loss of compartmental structural compliance. The prespecified reporting boundary of CDI greater than 0.5 corresponds algebraically to more than 55.6% loss of local structural compliance within this model. It has no established correspondence to histological injury, oxygenation, biomarker concentration, mortality, or a clinical diagnosis. Compartments crossing this boundary are therefore described as exceeding the model’s internal high-damage threshold rather than as clinically or functionally lost tissue.

Preserving both exposure and tissue state is necessary to interpret the mechanical economy of a ventilator adjustment. A reduction in subsequent TASH or SASH accumulation may indicate that a compartment has stabilized, but it may also occur because the compartment has stiffened, closed, or ceased to participate mechanically. Conversely, recruitment may increase the exposure received by a compartment because it has re-entered ventilation while its modeled tissue state remains comparatively preserved. FAAST retains both layers so that an intervention is not judged solely by recruitment, airway pressure, or a single aggregated dose value.

Respiratory rate entered this implementation through the RACE event counter relative to a reference rate of 15 breaths/min. It did not independently scale the direct strain-and-stress pathway. The present results therefore do not support conclusions about tidal-volume–respiratory-rate tradeoffs at fixed minute ventilation. Regional exposure, tissue state, and recovery parameters are listed in Table S5. Complete dose equations, tissue-state updates, parameter rationale, provenance classifications, and sensitivity analyses are provided in Supplementary Sections S3 and S5–S8.

### 2.6. Ventilator Protocol, Comparators and Experimental Design

The experiments examined the mechanical economy of the breath from four related perspectives. PEEP influenced how much recruitable lung remained available at end expiration, while tidal volume determined the dose distributed among the compartments participating in the next breath. Respiratory rate and inspiratory time influenced cumulative exposure and the number of RACE events but were held constant within each experiment. This design isolated defined mechanical questions without implying that any ventilator setting acts independently of the others. Complete protocols and experiment specifications are provided in Supplementary Section S4.

#### 2.6.1. Common Ventilator Protocol and Safety Envelope

Simulations used an idealized reference patient with a predicted body weight of 70 kg. Experiments 1, 3, and 4 began with a tidal volume of 450 mL, approximately 6.4 mL/kg, a respiratory rate of 15 breaths/min, and an inspiratory time of 1.0 s. Experiment 2 used a tidal volume of 420 mL, or 6 mL/kg, a respiratory rate of 18 breaths/min, and an inspiratory time of 1.0 s. The canonical simulation horizon was 96 h. Experiment 2 used a 24-h horizon because it examined early divergence between global and regional measurements rather than longer-term strategy performance. State variables were updated at 0.1-h intervals using a representative breath, with cumulative exposures scaled to the number of breaths delivered during each interval.

Clinically realistic strategy comparisons incorporated an ARDSNet-derived pressure safety rail [1]. When plateau pressure exceeded 33 cm H_2_O, tidal volume was reduced in steps to a lower boundary of 390 mL in the 450-mL protocol. The trigger allowed a 10% tolerance above the conventional 30 cm H_2_O plateau target before automatic volume reduction. The tidal-volume floor was a protocol boundary rather than a simulated gas-exchange target. Because the model does not calculate carbon-dioxide production, physiological dead space, pH, or the respiratory rate required to maintain alveolar ventilation, it cannot determine whether further tidal-volume reduction would be clinically tolerable in an individual patient. The ventilator-protocol and controller parameters are summarized in Table S6.

FAAST was evaluated as an analytical layer operating within this constrained ventilator envelope. Any future clinical application would remain subordinate to oxygenation, carbon-dioxide clearance, acid–base status, airway-pressure limits, hemodynamic and right-ventricular tolerance, patient–ventilator interaction, and clinician judgment. Evidence of circulatory compromise would override a mechanically derived recommendation to increase or maintain PEEP. One-at-a-time sensitivity analysis of the provisional barrier and RACE-pathway parameters are reported in Table S7.

#### 2.6.2. Ventilator Strategies and Comparators

Experiment 1 included fixed PEEP values of 5, 8, 10, and 15 cm H_2_O. PEEP of 8 cm H_2_O approximated the central tendency reported in LUNG SAFE and served as the principal practice comparator [5]. PEEP of 5 cm H_2_O represented a deliberately under-recruited boundary, while 10 and 15 cm H_2_O provided progressively greater fixed recruitment. These arms were designed to isolate different end-expiratory lung states and were not intended to represent complete clinical protocols.

An aggressive aeration-targeted strategy provided a stronger conventional recruitment comparator. This arm began at PEEP 8 cm H_2_O and reassessed mean end-expiratory openness every 4 h. PEEP increased by 2 cm H_2_O when mean openness remained below 0.65, to a maximum of 22 cm H_2_O. End-expiratory openness served as a reduced-order aeration surrogate because oxygenation was not modeled. This strategy should therefore not be interpreted as a clinical oxygenation-response protocol.

The legacy dose-guided controller was retained as a negative analytical result. Every 6 h, it evaluated candidate PEEP values from 6 to 24 cm H_2_O and minimized the sum of the compartmental injury signals, with penalties for insufficient end-expiratory openness and PEEP above 18 cm H_2_O. Although the underlying quantities were calculated regionally, summation permitted improvement in several compartments to offset severe deterioration in others. The controller therefore reproduced the loss of spatial information that FAAST was intended to address.

The FAAST crossover rule was developed after this limitation became apparent. Every 6 h, it evaluated the same candidate PEEP range without combining regional states into a single weighted objective. At each PEEP, the model counted compartments with a RACE index greater than 0.05 and compartments with a suprathreshold stress ratio greater than 1. The selected PEEP was the lowest value at which the number of suprathreshold compartments equaled or exceeded the number of RACE-active compartments. The rule therefore identified a regional transition between unresolved instability and increasing overdistension.

The crossover rule was empirically defined after evaluation of the legacy controller and was not derived as a mathematical optimum from the damage equations. Its origin and dependence on the operational RACE and stress thresholds are disclosed explicitly. Both thresholds were varied in Supplementary Section S7, with the complete results reported in Table S8. The crossover is evaluated here as a testable decision hypothesis, not as a validated patient-specific PEEP target.

#### 2.6.3. Experiment 1: Recruitment and Strategy Comparison

Experiment 1 used 60 virtual lungs sampled from the frozen phenotype and recruitability distributions with seed 20260710. Each lung was copied into all seven intervention arms so that every strategy began with the same severity, recruitability, opening pressures, barrier state, and structural compliance. The seven arms were fixed PEEP at 5, 8, 10, and 15 cm H_2_O; aggressive aeration-targeted recruitment; the legacy summed-dose controller; and the FAAST crossover. Each copy was ventilated independently for 96 h with the pressure safety rail active. The principal outcome was percentage loss of modeled structural compliance. Mean PEEP, plateau and driving pressures, phenotype-specific results, and the direction of the paired comparisons were also reported.

#### 2.6.4. Experiment 2: Regional Observability Under Identical Ventilation

Experiment 2 examined whether globally acceptable ventilator measurements could conceal different regional mechanical states. Four prescribed conditions were used: a preserved-mechanics reference and mild, intermediate, and severe injured-lung phenotypes, with baseline respiratory-system compliances of approximately 90, 45, 35, and 25 mL/cm H_2_O, respectively. Every phenotype received identical ventilation for 24 h: tidal volume 420 mL, PEEP 10 cm H_2_O, respiratory rate 18 breaths/min, and inspiratory time 1.0 s.

The global observations were respiratory-system compliance, plateau pressure, and driving pressure. Regional outputs included tidal-volume concentration, effective stress, the worst-compartment stress ratio, SASH, and CDI. Plateau pressure below 28 cm H_2_O and driving pressure below 15 cm H_2_O defined a prespecified descriptive no-action band. These values represented a clinically familiar range in which global mechanics alone might not prompt an immediate change; they were not treated as biological safety thresholds. Experiment 2 was an observability analysis rather than a comparison of treatment efficacy.

#### 2.6.5. Experiment 3: Recruitment–Overdistension Crossover

Experiment 3 characterized the transition from residual RACE to increasing regional overdistension across PEEP values from 4 to 24 cm H_2_O in 1-cm H_2_O increments. At each PEEP, the model recorded end-expiratory openness, recruitment, RACE-active and suprathreshold compartment counts, plateau and driving pressures, respiratory-system compliance, and the maximum regional stress ratio.

The crossover was first examined in representative moderate and severe phenotypes and was then applied, without changing the frozen model, to a fresh cohort of 50 virtual lungs generated with seed 77777. For each lung, the 96-h result at the crossover-selected PEEP was compared with fixed-PEEP strategies and with a retrospective model-defined optimum. The latter identified the PEEP producing the lowest completed 96-h structural-compliance loss and therefore required information unavailable at the bedside. It served only as an internal benchmark. The principal outputs were selected PEEP, modeled structural-compliance loss, the direction of the paired comparison with PEEP 8 cm H_2_O, and proximity to the retrospective optimum. Use of a fresh seed reduced dependence on the original cohort but did not constitute external validation.

#### 2.6.6. Experiment 4: Interaction Between Recruitment and Tidal-Volume Reduction

Experiment 4 tested whether the modeled effect of tidal-volume reduction depended on the preceding recruitment state. Fifty virtual lungs generated with seed 20260710 were evaluated in a matched 2 × 2 factorial design. Each lung received four conditions: PEEP 8 cm H_2_O or its FAAST crossover PEEP, each combined with tidal volumes of 450 or 390 mL. Paired differences were used to estimate the recruitment effect, the tidal-volume effect before recruitment, the tidal-volume effect after crossover recruitment, and the interaction between the two interventions. A positive interaction indicated that tidal-volume reduction produced a larger modeled benefit after the regional recruitment state had changed.

The factorial experiment was performed in two forms. The protocol-realistic analysis retained the plateau-pressure safety rail. This allowed the assigned tidal volume to fall when plateau pressure became excessive, although it also permitted the two low-PEEP conditions to converge when both reached the 390-mL floor. A mechanism-isolation analysis then disabled the safety rail so that each cell retained its assigned tidal volume. This preserved the factorial contrast but permitted clinically unacceptable pressures in the low-PEEP, higher-tidal-volume condition. The rail-disabled analysis was used only to examine mechanism; no clinical recommendation was derived from it.

The primary outcomes were paired differences in modeled structural-compliance loss. Worst-compartment stress ratio, worst and mean CDI, and the number of compartments exceeding the internal CDI reporting boundary were secondary outputs. These measures were included to distinguish broad preservation of regional participation from redistribution of injury into a smaller number of compartments.

#### 2.6.7. Descriptive Analysis

Once each virtual lung was generated, its mechanical simulations were deterministic. The study therefore did not use *p* values, confidence intervals, hazard ratios, or survival analysis as evidence of treatment effect. Results were summarized using medians, interquartile ranges where informative, paired within-lung differences, and the proportion of matched cases in which one strategy produced a lower modeled outcome than another.

These proportions describe consistency within the simulated cohorts and are not estimates of clinical response probability. The results are model-conditional mechanical predictions. External evaluation using patient waveforms, regional imaging, gas-exchange measurements, biomarkers, hemodynamic observations, and clinical outcomes will be required to determine whether the predicted regional states correspond to patient physiology and can support prospective ventilator guidance.

### 2.7. Generative AI Assistance

Generative AI-assisted tools from OpenAI (ChatGPT-5.5 Sol and Codex) and Anthropic (Claude Sonnet 5) were used to assist with Python 3.12.3 code drafting, debugging, consistency review, and editorial revision. The author defined the hypotheses, model architecture, equations, parameter boundaries, correspondence criteria, experimental designs, and interpretation; reviewed the resulting code and text; verified the cited sources; and assumes full responsibility for the work. All reported numerical results were produced by executing the frozen deterministic Python implementation and were not generated by a language model.

## 3. Results

### 3.1. Controller-Blind Correspondence Testing

The frozen phenotype model satisfied all nine prespecified correspondence gates (Table 2). Two construction changes were required: recruitability was changed from a symmetric to a right-skewed distribution, and an upper boundary was placed on the rate of structural-compliance decline. Both changes were made to satisfy the declared literature anchors before either dose-guided controller was tested. The gate definitions and tolerances were not subsequently changed.

### 3.2. Experiment 1: Recruitment and Controller Behavior

Experiment 1 demonstrated a nonlinear relationship between recruitment and modeled structural preservation. Across the fixed-PEEP arms, median structural-compliance loss at 96 h decreased from 68.8% at PEEP 5 cm H_2_O to 61.4% at PEEP 8, 46.5% at PEEP 10, and 39.3% at PEEP 15. Within this range, recruitment was the dominant modeled injury lever.

Fixed PEEP of 5 cm H_2_O produced a median plateau pressure of 81.8 cm H_2_O. This was not an isolated pressure transient. The tidal-volume safety rail was activated in 56 of 60 virtual lungs, but the 390-mL floor left no further volume-reduction option while PEEP remained fixed. As end-expiratory openness and structural compliance declined, the prescribed breath was concentrated into a progressively smaller mechanically available lung and driving pressure continued to rise.

This extreme result was retained because it is the direct output of the prototype and identifies failure of the imposed low-recruitment strategy. It should not be interpreted as a precise prediction of a clinically sustained plateau pressure. A patient developing comparable mechanics would require immediate intervention rather than continued ventilation at fixed PEEP 5. The current prototype contains no subsequent rescue escalation or termination rule and therefore continues to calculate the mechanical trajectory after the prescribed strategy has become untenable.

The adaptive strategies showed the second part of the tradeoff (Figure 2; Table 3). The aggressive aeration-targeted comparator reached a mean PEEP of 20.2 cm H_2_O and produced 52.7% median structural-compliance loss. The legacy controller, which minimized the sum of compartmental injury signals, reached a mean PEEP of 18.2 cm H_2_O and produced 43.2% loss. The FAAST crossover preserved the competing regional states rather than summing them into a single objective; it selected a mean PEEP of 13.5 cm H_2_O and produced 40.7% loss.

PEEP values are cohort medians of each virtual lung’s 96-h time-averaged PEEP. Plateau-pressure values are cohort medians of each virtual lung’s 96-h median ($P_{plat}$). Structural loss is the median percentage reduction in modeled structural compliance at 96 h. Differences versus PEEP 8 are differences between strategy medians, expressed in percentage points. The PEEP 5 arm was retained as a low-recruitment boundary challenge; its extreme plateau pressure represents continued calculation after the tidal-volume floor had been reached and indicates protocol failure rather than a clinically sustainable strategy.

The legacy controller is retained as a negative result. Although it calculated injury signals by compartment, summation allowed improvement in some regions to offset persistent RACE or overdistension in others. The aggressive aeration-targeted strategy likewise acted on a whole-lung mean and continued recruitment into a higher-PEEP state. Both approaches lost the regional competition that the compartmental crossover was designed to preserve.

FAAST produced less modeled structural loss than PEEP 8 in 100% of matched cases and less than aggressive aeration-targeted recruitment in 98% (Figure 3). These percentages describe consistency within the deterministic cohort and are not estimates of clinical treatment effect. Fixed PEEP 15 produced the lowest median loss among the tested strategies, at 39.3%, compared with 40.7% under the FAAST crossover. Experiment 1 therefore does not establish superiority over every fixed-PEEP strategy. It shows that a compartmental rule approached the most favorable fixed-arm result without prespecifying one PEEP for every lung and avoided the higher-PEEP behavior produced by the aggregate-guided strategies.

### 3.3. Experiment 2: Regional Injury Under Globally Acceptable Ventilation Settings

With ventilation held constant, only phenotype changed. In the prescribed severe baby-lung phenotype, six mechanically participating compartments received approximately 87% of each breath, while refractory compartments received little tidal volume.

In the mild and intermediate phenotypes, plateau pressure was 19.8 and 23.0 cm H_2_O and driving pressure was 9.8 and 13.0 cm H_2_O. Despite these reassuring global values, worst-compartment stress ratios were 1.40 and 1.58, and model-internal CDI reached 0.062 and 0.117 at 24 h (Table 4; Figure 4).

Within the severe phenotype, regional stress ratio ranged from 0.46 to 1.82 under the same breath, with more than a tenfold difference in accumulated strain-energy burden. A 10% tidal-volume reduction moved the worst compartment from 2.02 to 1.95 but did not bring it below the model threshold. The model therefore attributed risk not only to breath size, but to its regional distribution.

### 3.4. Experiment 3: Compartmental PEEP Crossover

After the summed-dose controller failed, we defined a compartmental crossover: raise PEEP while cycling compartments outnumber suprathreshold compartments and stop at the lowest PEEP where suprathreshold compartments equal or exceed cycling compartments. The rule is a testable decision hypothesis, not a proven optimum.

Post hoc threshold sensitivity showed that exact selection was threshold-dependent (Table 5; Figure 5). Across a tenfold decrease and increase in the cycling cutoff and stress-ratio cutoffs of 0.8, 1.0, and 1.2, median selected PEEP ranged from 9 to 16 cm H_2_O and median modeled loss from 39.1% to 52.4%. Nevertheless, every combination reduced median loss versus PEEP 8 by 9.6–22.9 points and was favorable in 92–100% of matched cases. Thus, direction was robust, but precise PEEP selection was not threshold-invariant.

In the independent cohort, the nominal crossover selected median PEEP 12, compared with 15 for the retrospective model-defined optimum. Median loss was 42.5% versus 62.0% at PEEP 8 and 38.9% at the model-defined optimum. The crossover was favorable to PEEP 8 in all matched cases but fell within two loss points of the model optimum in 30%, confirming that it should not be described as an exact optimizer.

Current global bedside measures cannot directly count cycling and suprathreshold regions. The proposed clinical role is therefore to add an estimated regional state to established pressure, volume, gas-exchange, and hemodynamic signals, not to replace them.

### 3.5. Experiment 4: Recruitment Amplifies the Tidal-Dose Lever

The proposed sequence, stabilize recruitable lung, then reduce residual cyclic dose, predicts that tidal-volume reduction should have greater modeled effect after recruitment. We tested that prediction in a matched 2 × 2 factorial.

With the clinical safety rail active, low-PEEP plateau pressure triggered tidal-volume reduction to 390 mL in 78% of virtual patients, causing the two low-PEEP cells to converge. This run reflects protocol behavior but cannot isolate the tidal-volume effect.

The mechanism-isolated run disabled the safety rail only to preserve the assigned 450-versus-390-mL contrast. It is not a clinically acceptable ventilation scenario and is interpreted solely as a mechanistic probe.

In paired within-patient analysis, tidal-volume reduction produced a median 1.2-point benefit at practice PEEP and a 5.9-point benefit after FAAST recruitment (Table 6; Figure 6). The median recruitment effect was 19.1 points, the combined effect 25.4 points, and the positive interaction 4.4 points; the interaction was positive in 92% of patients.

The model therefore supports a conditional sequence rather than a universal directive: while recruitment-derecruitment dominates, reducing tidal volume has limited leverage; after more units stabilize, the residual injury becomes more responsive to tidal-volume reduction.

### 3.6. The Ceiling: Mitigation, Not Protection

No tested PEEP and tidal-volume combination eliminated modeled regional injury. Even with the crossover and tidal volume at 390 mL, the worst compartment remained suprathreshold in every virtual patient.

Worst-compartment stress ratio was higher under FAAST than under practice (1.81 versus 1.49), reflecting additional static strain from PEEP (Table 7). The difference appeared in the surrounding compartment population rather than in the single worst region.

Worst-compartment CDI remained approximately 0.91 in every arm. The number of compartments crossing the prespecified model-internal CDI > 0.5 cutoff fell from 17 of 20 with practice ventilation to 8 of 20 with FAAST plus tidal-volume reduction.

FAAST was therefore not a peak-stress minimizer. In this simulation, it redistributed regional burden: higher stress in the worst compartment coexisted with lower mean CDI and fewer compartments above the model cutoff. The cutoff does not identify clinical loss of function and should not be interpreted as a patient outcome. This tradeoff is inherent to mechanical ventilation of a heterogeneous injured lung. FAAST does not introduce the tradeoff; it makes the tradeoff explicit.

### 3.7. Robustness and Sensitivity

Across the original seven parameter configurations, severity ordering, the early injury lag, and the directional advantage of the crossover were preserved; FAAST was favorable versus PEEP 8 in 100% and versus aggressive recruitment in 95–98% of cases. Operational-threshold sensitivity changed median selected PEEP from 9 to 16 cm H_2_O but preserved lower median loss versus PEEP 8 in every configuration. Removing the provisional strain-history modifier reduced median FAAST loss from 40.7% to 39.6%; FAAST remained favorable versus PEEP 8 in 100% and aggressive recruitment in 96.7% of cases. However, the legacy-versus-crossover ordering reversed (38.3% versus 39.6%), showing that this specific controller comparison depends on the fatigue-modifier assumption. Complete results of the strain-history susceptibility-modifier ablation are provided in Table S9.

The public files, scripts, seeds, and execution resources supporting reproducibility are listed in Table S10.

## 4. Discussion

### 4.1. Principal Findings

This study asked a practical question: after conventional lung-protective boundaries have been applied, what is happening within the regions of the lung that actually receive the breath? To examine that question, we applied the FAAST analytical framework to a frozen, twenty-compartment representation of heterogeneous acute lung injury. The model was anchored to clinically recognizable compliance phenotypes and then used as a controlled substrate through which the regional consequences of alternative ventilator patterns could be compared.

Several findings emerged. First, globally acceptable plateau pressure and driving pressure could coexist with suprathreshold loading in individual compartments. Second, regional information could be calculated and still lose its value if opposing injury mechanisms were combined into a single whole-lung objective. Third, the balance between repeated alveolar collapse and expansion (RACE) and overdistension changed progressively with PEEP, creating a model-derived crossover rather than a universally protective setting. Finally, the effect of tidal-volume reduction depended on recruitment state: smaller breaths had limited modeled leverage while RACE remained dominant, but became more effective after a larger fraction of recruitable lung had been stabilized.

The principal result is therefore not a new universal PEEP target. FAAST does not introduce the recruitment–overdistension tradeoff; it makes explicit a tradeoff already being managed at the bedside without regional observability. The object of ventilator-setting personalization should therefore extend beyond the setting itself to include the regional biomechanical state upon which the complete ventilator pattern acts. PEEP, tidal volume, respiratory rate, inspiratory time, and flow remain global controls, but their consequences depend on how much lung is open, how that participating lung is distributed, and which regions remain unstable, overdistended, or mechanically vulnerable.

These findings are model-conditional. The simulation is a literature-anchored prototype testbed, not an externally validated patient-specific predictor. Within that boundary, it supports a coherent and falsifiable hypothesis: regional mechanical intelligence may reveal clinically relevant tradeoffs that cannot be recovered from whole-lung measurements alone.

### 4.2. The Information Lost Through Aggregation

The failure of the legacy dose-minimizing controller was especially informative. That controller was not blind to compartmental mechanics. It calculated dose regionally, but then minimized the sum of those regional values. Summation allowed persistent RACE in some compartments to trade numerically against overdistension in others, even though the two states represent different mechanisms and may require opposing ventilator responses. The resulting controller moved toward a high-PEEP regime similar to the aggressive aeration-targeted strategy.

This finding does not diminish plateau pressure, driving pressure, respiratory-system compliance, oxygenation, or mechanical power. These measurements remain indispensable bedside signals and necessary safety boundaries. Their limitation is one of resolution. Each reduces the behavior of a mechanically heterogeneous organ to a scalar value, and the same global value may arise from very different distributions of regional stress, deformation, and instability.

Experiment 2 demonstrated this observability problem from the patient side. With the ventilator pattern held constant, the mild and intermediate phenotypes produced plateau and driving pressures that would not ordinarily trigger concern, while the model estimated suprathreshold loading in their most exposed compartments. In the severe baby-lung phenotype, approximately 87% of tidal volume was directed into only six mechanically participating compartments. This is consistent with the baby-lung concept and with the clinical observation by Terragni and colleagues that tidal hyperinflation may persist despite ventilation at 6 mL/kg predicted body weight and plateau pressure below 30 cm H_2_O [13,16].

The implication is not that global measurements are wrong. They answer a different question. They describe the breath delivered at the airway and the response of the respiratory system as a whole, whereas FAAST asks how that breath may be distributed after it encounters a heterogeneous lung. The proposed value of regional analysis is the additional information it may recover before regional deterioration becomes visible in whole-lung mechanics.

### 4.3. RACE, Recruitment, and the PEEP Tradeoff

Repeated alveolar collapse and expansion is a clinically recognizable form of regional instability. Direct microscopy in an experimental acute lung-injury model demonstrated that surfactant deactivation increased RACE and that PEEP could mechanically stabilize affected alveoli across a range of tidal volumes [10]. Subsequent experimental work has shown how recruitment can focus injurious power at an air–liquid interface and how repeated interfacial events can erode epithelial barrier function before abrupt failure becomes apparent [9,24]. The present model does not reproduce those events at cellular resolution; its compartmental RACE measure is a reduced-order proxy for the repeated opening and closing of mechanically unstable lung units.

PEEP is therefore neither intrinsically protective nor intrinsically injurious. At insufficient PEEP, recruitable compartments remain unstable and repeatedly close and reopen. As PEEP rises, some of those compartments become stably available to receive ventilation, increasing the effective size of the participating lung [28]. Beyond that benefit, however, further pressure increasingly loads compartments that are already open and shifts the regional balance toward overdistension. The relevant question is not whether PEEP should be minimized or maximized, but where recruitment benefit begins to be overtaken by regional cost.

The fixed-PEEP-5 arm illustrates the lower boundary of that relationship. Its 96-h median plateau pressure of 81.8 cm H_2_O is an extreme model output, but it is not a transcription error. With little lung remaining open, the prescribed tidal volume was forced into a progressively smaller mechanically participating fraction. The ARDSNet-derived safety rail reduced tidal volume to its modeled floor in most virtual patients, but fixed low PEEP provided no recruitment or rescue mechanism. The simulation therefore continued into a mechanically untenable state. This value should not be interpreted as a pressure likely to be tolerated in clinical practice; it identifies protocol failure within the model and demonstrates the need for an explicit clinician override or terminal failure condition in later implementations.

At the opposite boundary, aggressive aeration-targeted recruitment selected a mean PEEP of 20.2 cm H_2_O and produced greater modeled structural loss than either fixed PEEP 15 or the FAAST crossover. The legacy summed-dose controller behaved similarly, although less severely. These findings reproduce the expected nonlinear character of PEEP: inadequate recruitment concentrates the breath, while excessive recruitment exchanges RACE for overdistension and sustained tonic loading.

The crossover was introduced to preserve that distinction. It selected the lowest PEEP at which suprathreshold compartments equaled or outnumbered RACE-active compartments. This rule should not be presented as a proven optimum. Its exact PEEP was sensitive to the operational thresholds, and in the fresh virtual cohort it did not consistently reproduce the retrospective model-defined optimum. Fixed PEEP 15 also produced slightly less median structural loss than the crossover in Experiment 1. The importance of the crossover is therefore not superiority over every fixed setting; it is that it approached the most favorable fixed-PEEP result without assigning the same PEEP to every phenotype and stopped before the higher-pressure behavior produced by the two aggregated strategies.

### 4.4. The Mechanical Economy of the Complete Breath

FAAST is not a PEEP-selection model alone. It evaluates the complete ventilator pattern as it acts upon a defined lung state. PEEP establishes the end-expiratory mechanical field, but tidal volume, respiratory rate, inspiratory time, and flow determine how repeatedly and how intensely that field is loaded. A ventilator adjustment cannot therefore be interpreted in isolation from the lung receiving it or from the other components of the breath.

Experiment 4 illustrates this interaction. When the lung remained under-recruited, reducing tidal volume from 450 to 390 mL produced only a small modeled benefit because RACE remained the dominant insult. After more recruitable compartments were stabilized, the same tidal-volume reduction produced a larger effect. This does not establish a universal sequence of raising PEEP before lowering tidal volume. It suggests instead that recruitment state alters the effectiveness of subsequent dose reduction. Smaller breaths cannot fully compensate for a lung that continues to close and reopen, just as recruitment cannot justify unrestricted tidal volume once a larger participating surface has become available.

The regional dose channels further clarify this relationship. Time-Accumulated Stress History (TASH) describes sustained regional wall-stress exposure, whereas Strain-Accumulated Strain History (SASH) describes accumulated cyclic deformation-energy burden. Under FAAST-guided recruitment, tonic stress exposure increased in some compartments because more PEEP was applied, yet cyclic burden fell in previously unstable regions and the load was shared across a larger participating population. A rise in TASH accompanied by a fall in mean Continuum of Damage Index (CDI) is therefore not contradictory. Delivered exposure, its regional distribution, and its tissue-state consequence are related, but they are not interchangeable.

This is the mechanical economy FAAST is intended to examine: how much viable lung can be made stably available for ventilation, and at what regional mechanical cost. The most economical breath is not necessarily the breath with the lowest airway pressure, lowest PEEP, or lowest value of any single dose channel. It is the ventilator pattern that supports gas exchange while preserving the largest feasible mechanically participating lung surface and limiting avoidable regional injury.

The model also makes clear that this economy has a ceiling. No tested combination eliminated regional injury, and the worst compartment remained suprathreshold in every virtual patient. FAAST did not minimize the stress or CDI of that single compartment. Its modeled effect appeared in the surrounding population, where mean injury and the number of compartments crossing the internal CDI threshold were reduced. The appropriate claim is therefore mitigation, not absolute protection. Some regions may already be mechanically lost or may remain vulnerable under every clinically reasonable global setting; the objective is to prevent that burden from spreading unnecessarily through the remaining lung.

### 4.5. Clinical Translation and Safety Boundaries

FAAST is proposed as a clinician-in-the-loop analytical layer, not an autonomous ventilator controller. Mechanical delivery remains global. The intended contribution is to estimate the regional response to that delivery and place it beside the physiologic information clinicians already use.

A practical implementation would operate as a repeated clinical loop. A phenotype-conditioned patient-state model would first be initialized from available mechanics and, where possible, imaging or electrical impedance tomography. Ventilator settings and waveforms would then be mapped through that state to estimate the participating lung fraction, RACE-active and overdistended regions, regional dose distribution, and tissue-state trajectory. These estimates would be displayed alongside plateau pressure, driving pressure, compliance, gas exchange, hemodynamics, and the patient’s clinical course. The clinician could then consider a global change in PEEP, tidal volume, rate, inspiratory time, or flow and reassess its predicted and observed consequences.

This workflow is intended to add intelligence to established lung-protective care, not to replace it. No FAAST-derived mechanical recommendation should override inadequate oxygenation, carbon-dioxide clearance, unacceptable pH, circulatory compromise, right-ventricular intolerance, or clinical judgment. The present implementation cannot adjudicate those competing constraints because it does not model gas exchange or hemodynamics. In particular, a mechanically favorable PEEP estimate must be rejected when the patient demonstrates cardiovascular or other physiologic intolerance.

The most appropriate first clinical use is therefore shadow-mode evaluation. FAAST outputs could be generated from archived or live ventilator waveforms without influencing care and compared with simultaneous or subsequent regional imaging, compliance evolution, gas-exchange and hemodynamic trajectories, biological injury markers, and clinical deterioration or recovery. This would test whether its estimated regional states correspond to events occurring in patients before any interventional recommendation is considered.

A computational model can contribute to clinical translation before it becomes a clinical device. It can expose assumptions, test whether proposed mechanisms behave coherently, identify contradictions in current decision logic, and generate predictions that can be measured and falsified. Direct patient-specific guidance, however, requires independent calibration, uncertainty quantification, prospective validation, and integration with the full physiologic safety environment.

### 4.6. Limitations and Development Pathway

The evidence tier of this study is in silico mechanistic prediction. The crossover, regional stress ratios, TASH, SASH, CDI, structural-compliance loss, and matched-strategy differences are calculated outputs, not measured patient outcomes. They should not be interpreted as estimates of clinical treatment effect, mortality, histologic injury, or individual prognosis.

A reduced-order model was used deliberately at this stage. Routine clinical datasets do not contain simultaneous measurements of regional stress, strain, RACE, epithelial barrier state, and evolving tissue injury with which the entire FAAST architecture could be directly fitted. A controlled phenotype-conditioned substrate therefore allowed the analytical engine to be tested against known mechanical relationships before patient-specific inference was attempted. This developmental choice does not substitute for external validation; it defines the prototype step that precedes it.

The substrate itself contains important simplifications. Twenty parallel compartments cannot reproduce the full anatomical, spatial, temporal, and biological complexity of ARDS [29,30]. Phenotypes were initialized from published compliance distributions and held fixed rather than being allowed to evolve. The model does not represent independent inflammatory progression, pulmonary vascular behavior, gas exchange, dead-space fraction, spontaneous effort, flow limitation, circuit mechanics, right-ventricular function, renal perfusion, sedation burden, or barotrauma outside its mechanical stress formulation. Respiratory-rate behavior was developed near 15 breaths/min, so rate–volume tradeoffs should not be generalized beyond that neighborhood.

The literature anchors also serve different purposes. Panwar and colleagues’ LUNG SAFE analysis supplied the clinical compliance structure used to create the acute lung-injury phenotypes [17]. The prolonged stability described in Park and colleagues’ ventilator-dependent amyotrophic lateral sclerosis cohort was used only as a conceptual homeostatic floor, demonstrating that prolonged mechanical ventilation need not produce inevitable modeled deterioration in the absence of an unstable injured substrate [21]. Neither source constitutes patient-level validation of FAAST.

Several model quantities remain provisional. CDI is a bounded internal state variable, and the cutoff of CDI > 0.5 corresponds algebraically to greater than 55.6% modeled local structural-compliance loss under the selected mapping. It does not correspond to a validated biological, histological, or clinical threshold. The strain-history modifier also has provisional kinetics, and the reversal of the legacy-versus-crossover ordering in the ablation analysis shows that this particular comparison depends on that assumption. The primary conclusions concerning regional heterogeneity, RACE–overdistension competition, and the limitations of aggregation did not depend on that ordering.

The crossover was developed after the summed-dose controller failed and was not derived as a mathematical optimum of the damage equations. Testing it with an independent random seed reduced dependence on the development cohort but did not provide external validation. Threshold sensitivity preserved its directional advantage over PEEP 8 while moving the selected median PEEP from 9 to 16 cm H_2_O. The crossover should therefore remain a candidate decision rule whose operational definitions must be identified empirically.

Experiment 4 presents an additional boundary. With the clinical safety rail active, low-PEEP patients frequently reached the tidal-volume floor, causing the low-PEEP factorial cells to converge. The rail-off analysis preserved the assigned tidal-volume contrast but allowed ventilation that would not be acceptable clinically. Its interaction estimate is therefore a mechanism-isolation result, not a proposed treatment protocol. Similarly, the extreme fixed-PEEP-5 trajectory indicates where a future model should declare ventilatory failure or require clinician rescue rather than continue calculating an untenable state.

The next developmental step is not additional unanchored complexity. It is external testing against independent observations. Priority targets include synchronized ventilator waveforms with electrical impedance tomography or computed tomography, regional ventilation and recruitment behavior, compliance trajectories, gas-exchange and hemodynamic responses, and biological markers of epithelial or endothelial injury such as sRAGE or surfactant protein D where available. These data can determine whether the modeled regional states are identifiable, whether CDI and fatigue-related parameters can be calibrated to measurable endpoints, and whether a more complex formulation improves correspondence enough to justify its added parameters.

Prospective evaluation should follow only after that correspondence has been established. The relevant clinical question will not be whether FAAST identifies a universally optimal setting, but whether FAAST-informed interpretation helps clinicians preserve regional participation, recognize occult mechanical deterioration, and adjust the complete ventilator pattern without compromising gas exchange or circulation.

## 5. Conclusions

The ARDS Network established that limiting whole-lung mechanical burden improves outcomes. FAAST extends that protective logic by asking where the remaining burden is distributed after global safety targets have been applied. In this frozen twenty-compartment model, globally acceptable ventilation could coexist with regional suprathreshold loading, while aggregation concealed the competing effects of RACE, recruitment, and overdistension.

These simulations support a bounded but clinically important hypothesis: regional mechanical intelligence may help clinicians understand how PEEP, tidal volume, respiratory rate, inspiratory time, and flow act together upon a heterogeneous lung. FAAST does not establish clinical efficacy, provide a validated patient-specific PEEP target, or make ventilation non-injurious. It offers a testable pathway toward a more informed mechanical economy of the breath—one that seeks to preserve the greatest feasible participating lung while limiting avoidable regional injury and respecting the physiology of the whole patient.

## Figures and Tables

**Figure 1:**
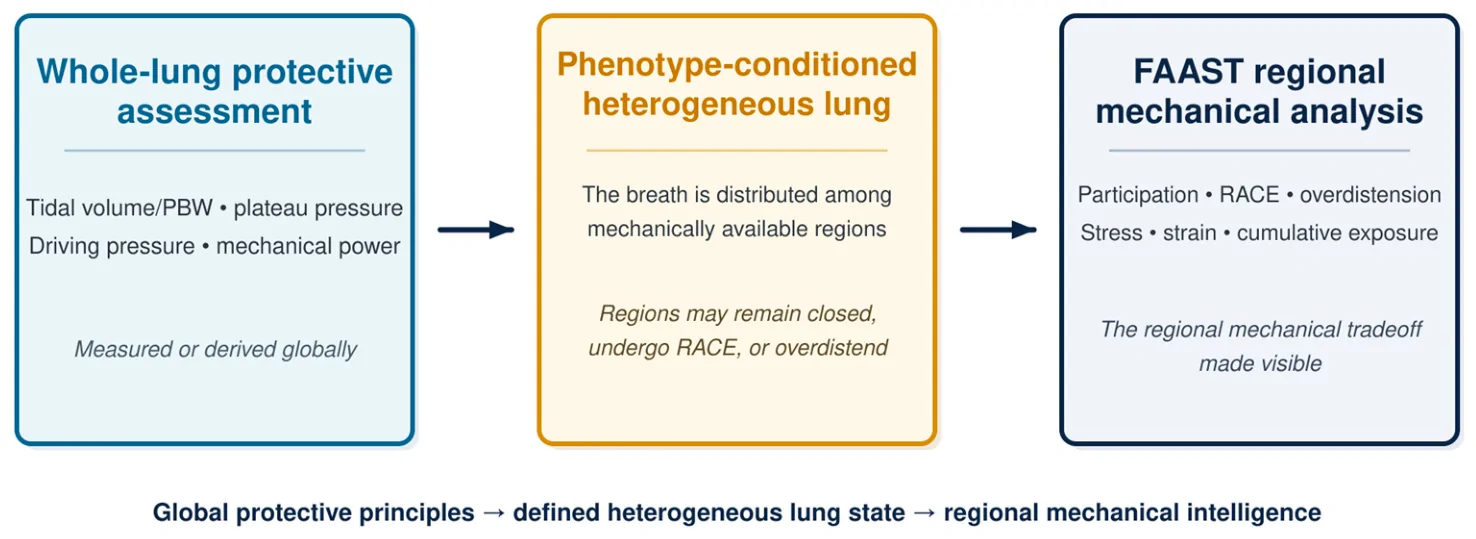
Conceptual extension from whole-lung protection to phenotype-conditioned regional analysis. Conventional variables describe the global ventilator–respiratory system interaction. The heterogeneous injured lung redistributes the applied breath among regions with different mechanical states. FAAST estimates the resulting regional participation, repeated alveolar collapse and expansion (RACE), overdistension, and cumulative mechanical burden. It supplements established lung-protective ventilation and clinical judgment rather than replacing either.

**Figure 2:**
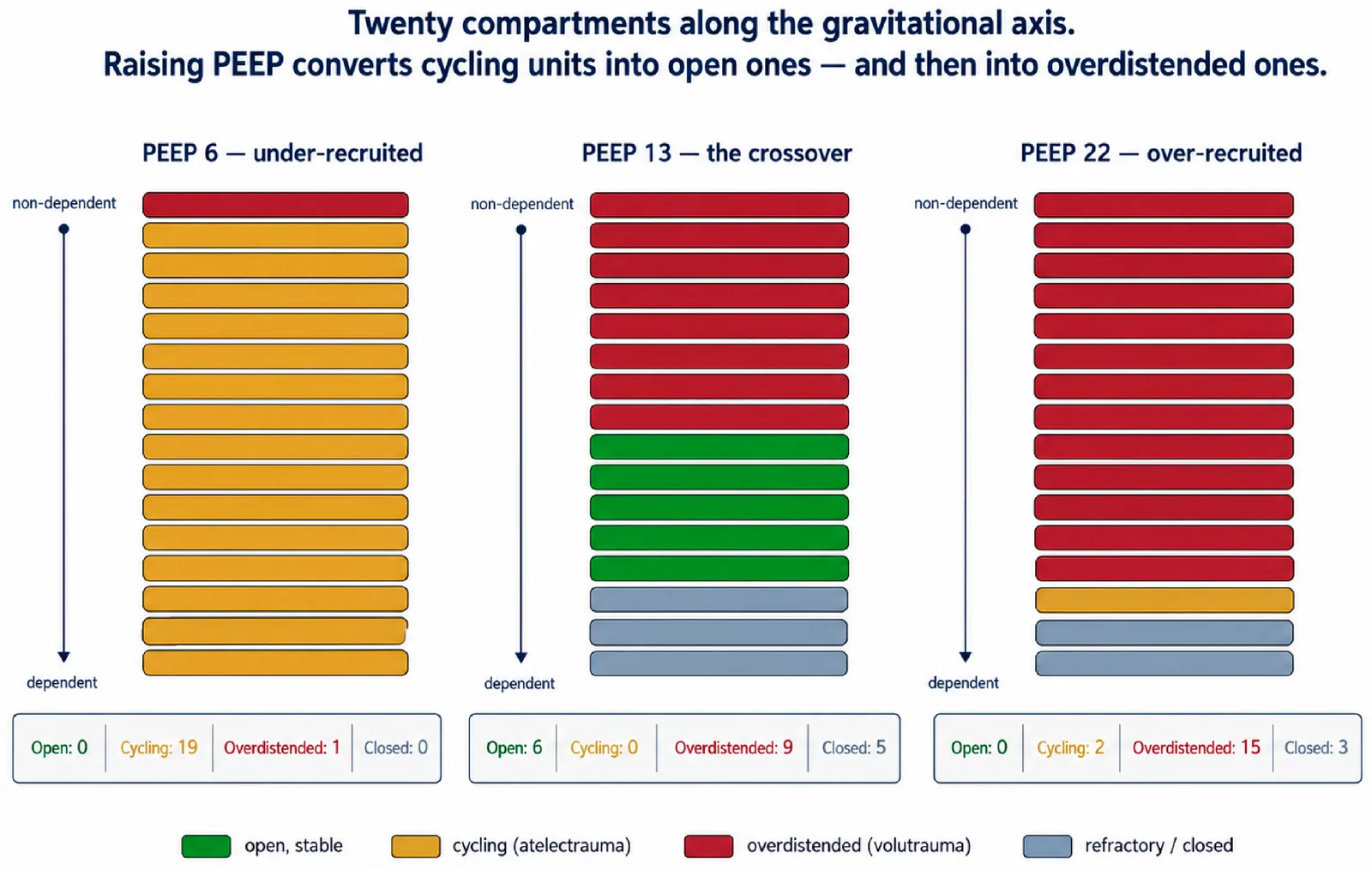
Compartmental transition across PEEP. Representative twenty-compartment model states are shown at PEEP 6, 13, and 22 cm H_2_O. At low PEEP, most compartments remain RACE-active; near the crossover, some compartments become open and stable while others become suprathreshold; at high PEEP, overdistension predominates. The illustration depicts a representative phenotype rather than cohort-median results and demonstrates why increasing PEEP cannot be interpreted as uniformly protective.

**Figure 3:**
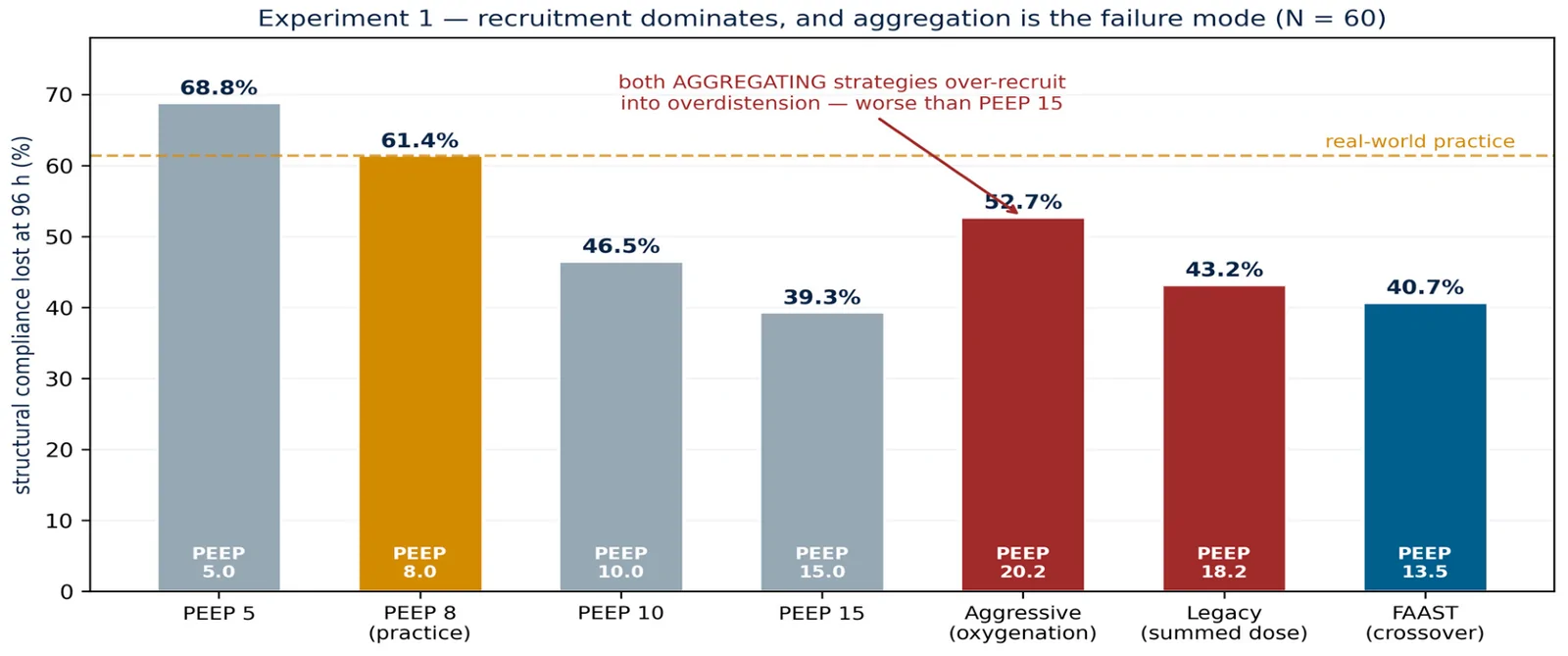
Experiment 1 strategy comparison. The x-axis presents the seven tested ventilation strategies; the fixed or mean PEEP associated with each strategy is shown within its bar in cm H_2_O. Gray bars denote the fixed-PEEP comparators, gold denotes the common-practice PEEP 8 comparator, red denotes the two aggregated-control strategies, and blue denotes the FAAST compartmental crossover. The dashed gold line marks the modeled structural-compliance loss under the common-practice PEEP 8 comparator (61.4%). Recruitment reduced modeled loss, but aggressive aeration-targeted (presumed oxygenation-guided) recruitment and the legacy summed-dose controller moved into a higher-PEEP overdistension regime. The compartmental crossover achieved similar modeled preservation to fixed PEEP 15 at a lower mean PEEP.

**Figure 4:**
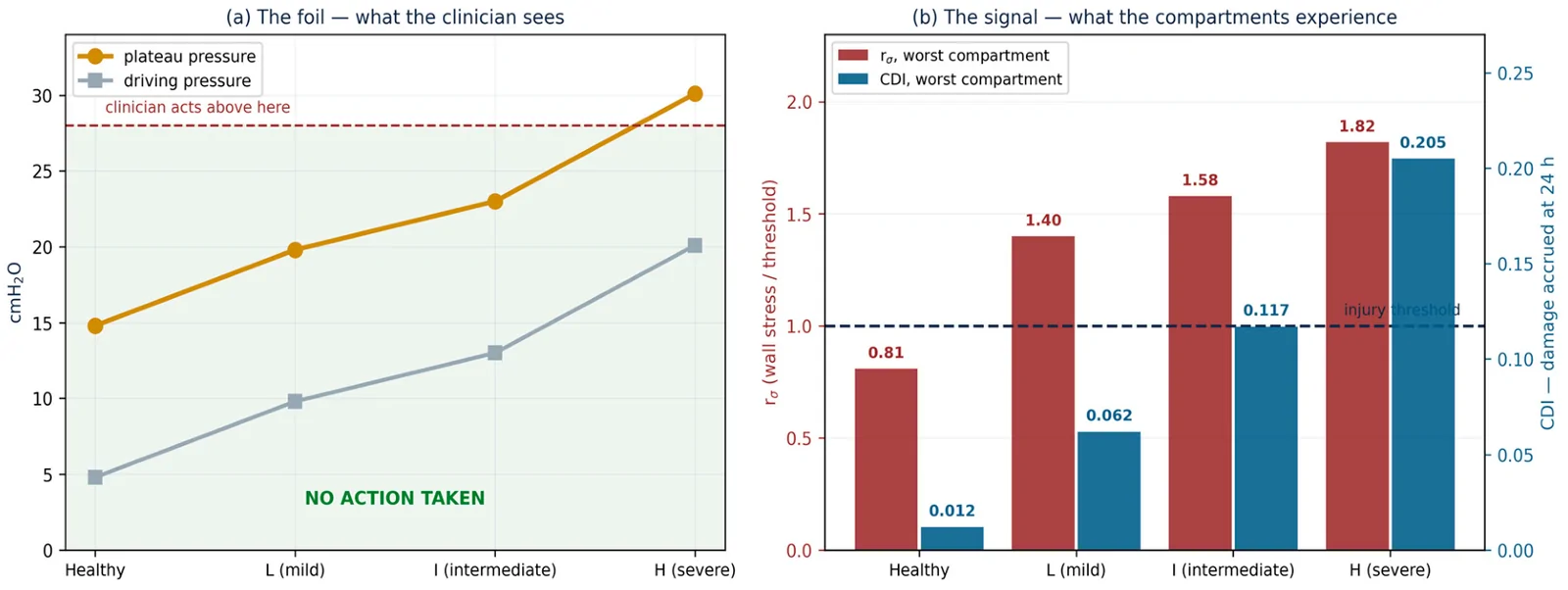
Experiment 2 regional observability. Under identical ventilation, global pressure and compliance could appear acceptable while worst-compartment stress ratio and CDI increased with phenotype severity. Panel (**a**) shows plateau pressure (gold) and driving pressure (gray). The red dashed line marks the prespecified plateau-pressure action threshold of 28 cm H_2_O, above which conventional global metrics would be expected to prompt clinical intervention. Panel (**b**) shows the worst-compartment suprathreshold stress ratio, rσ (red; left axis), and worst-compartment CDI at 24 h (blue; right axis). The blue dashed line at rσ = 1 marks entry into the model-defined suprathreshold loading regime; only the stress-ratio values are evaluated against this boundary, whereas CDI is read from the right axis. The separation between these thresholds illustrates how regional suprathreshold loading may remain occult while conventional global indicators have not yet prompted intervention. The figure depicts a model-predicted observability gap rather than measured patient injury.

**Figure 5:**
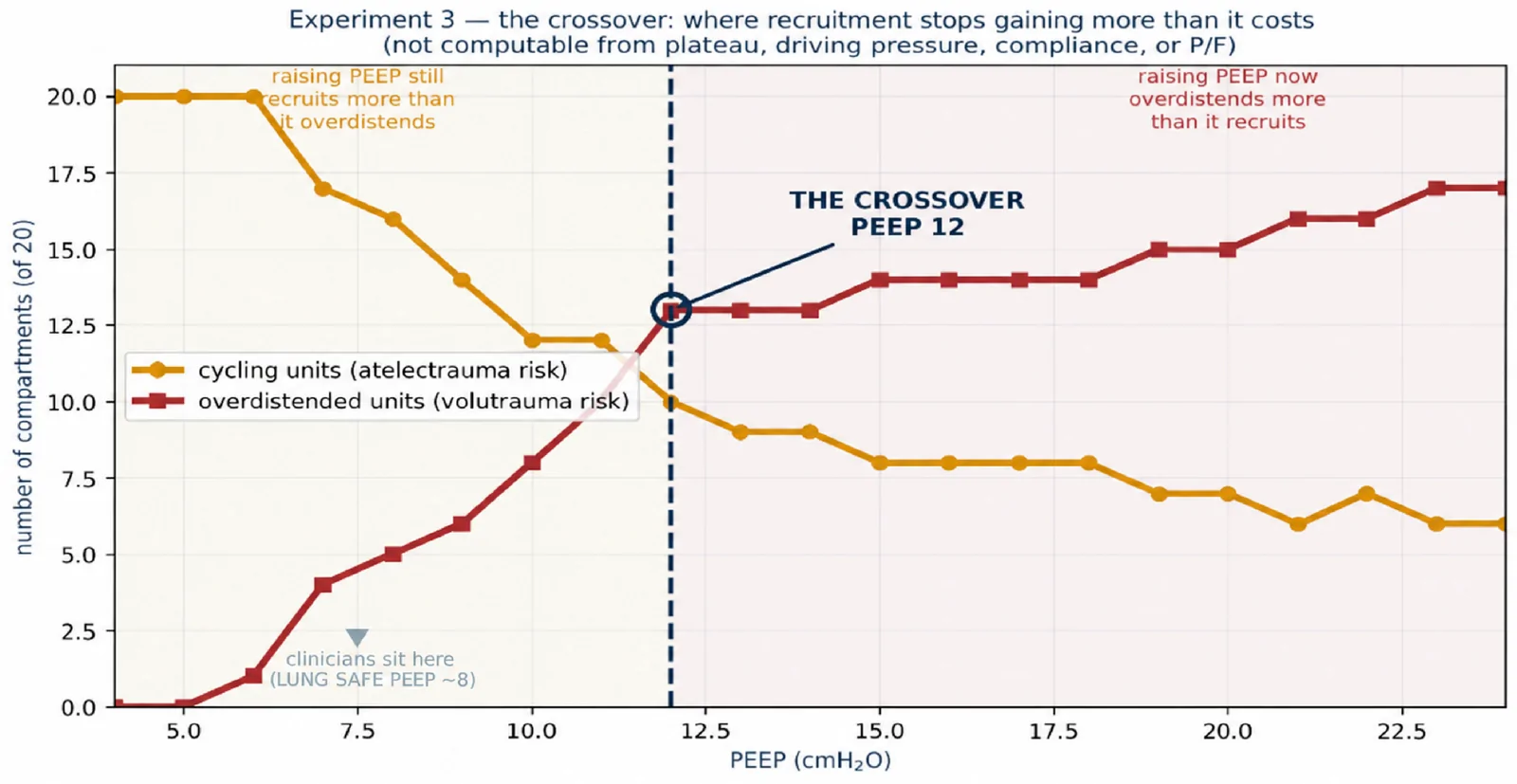
Experiment 3 compartmental PEEP crossover. At low PEEP, cycling compartments dominate; as PEEP rises, cycling decreases and overdistension increases. The black vertical dashed line marks the selected crossover at PEEP 12 cm H_2_O, the first tested PEEP at which the number of overdistended compartments equaled or exceeded the number of cycling compartments. This model-derived, threshold-dependent decision hypothesis requires external validation.

**Figure 6:**
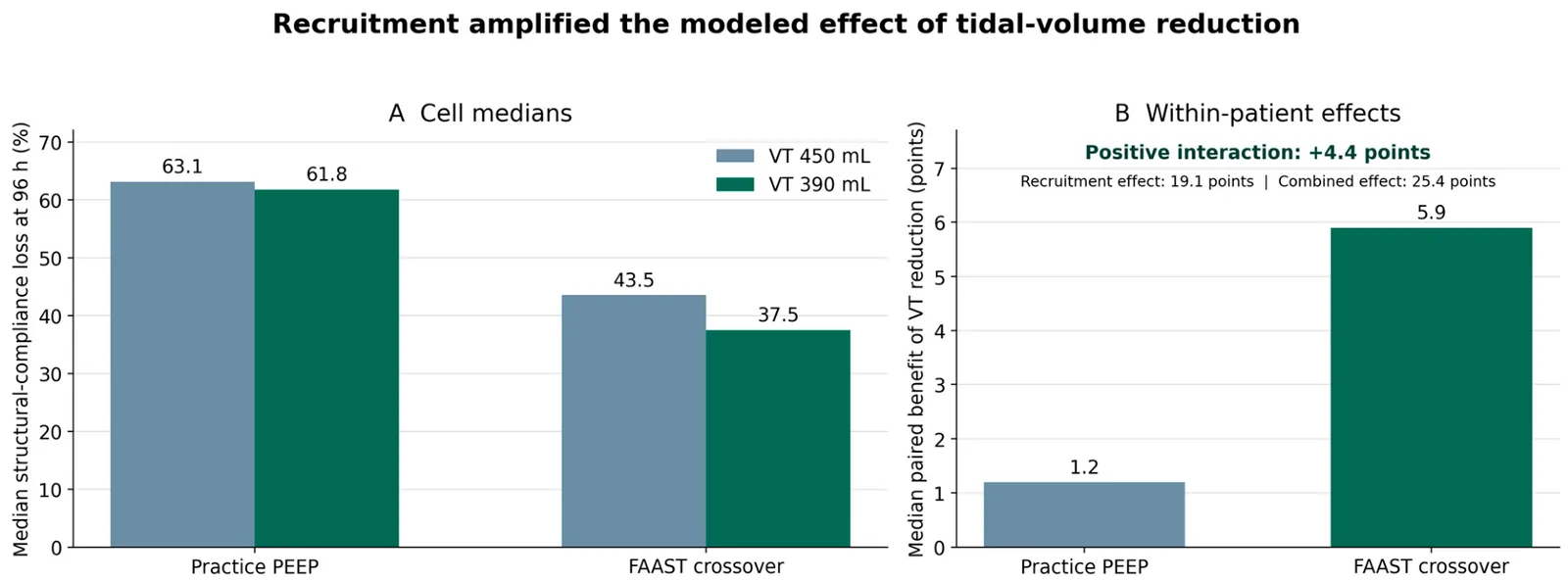
Experiment 4 interaction. Panel (**A**) shows cell medians; blue-gray bars represent a tidal volume of 450 mL, and green bars represent a tidal volume of 390 mL. Lower values indicate less modeled structural-compliance loss. Panel (**B**) shows the median paired within-patient benefit of reducing tidal volume from 450 to 390 mL; the blue-gray bar represents the effect at practice PEEP, and the green bar represents the effect after recruitment to the FAAST crossover. Tidal-volume reduction produced a 1.2-point benefit at practice PEEP and a 5.9-point benefit after FAAST recruitment, with a 4.4-point positive interaction. The safety rail was disabled for this mechanism-isolated run; therefore, the result is not a clinical protocol comparison.

**Table 1:** Experimental roadmap. Each experiment answers one clinically framed model question.

Experiment	Question	Design	Primary Model Output
1	Does aggregation change strategy ranking?	*N* = 60 matched; seven strategies; 96 h	PEEP and structural-compliance loss
2	Can acceptable global settings conceal regional burden?	Four prescribed phenotypes; fixed ventilation; 24 h	Worst regional stress ratio, SASH, and CDI
3	Can a compartment count locate a PEEP tradeoff?	PEEP 4–24; fresh *N* = 50 cohort	Selected PEEP and loss versus practice and model optimum
4	Does recruitment modify the tidal-volume effect?	Matched *N* = 50, 2 × 2 factorial	Paired effects and interaction

Abbreviations: *N*, number of virtual lungs; PEEP, positive end-expiratory pressure; SASH, Strain-Accumulated Strain History; CDI, Continuum of Damage Index.

**Table 2:** Controller-blind correspondence fence for the phenotype-conditioned lung model.

	Target	External Anchor	Tolerance	Model	Result
1	Phenotype distribution (mild/intermediate/severe)	Panwar and LUNG SAFE [5,17]	12%/14%/74%, sampling tolerance	11.5%/15.0%/73.5%	Pass
2	Recruitability distribution (mean/median)	Gattinoni et al. [22]	Mean 0.13; median 0.09	Mean 0.116; median 0.091	Pass
3	Severe-phenotype baseline mechanics	LUNG SAFE and Amato et al. [5,6]	Compliance 20–30 mL/cm H_2_O; ΔP 15–18 cm H_2_O	27.4 mL/cm H_2_O; 16.4 cm H_2_O	Pass
4	Onset of modeled injury acceleration	Caironi and Gattinoni et al. [23]	18 ± 11 h	20.0 h	Pass
5	Injury-trajectory form	Yamaguchi et al. [24]	Lag, acceleration, bounded plateau	Satisfied; peak decline at 20.6 h	Pass
6	Severity ordering	Clinical acute-lung-injury behavior	Greater deterioration, severe > mild	56.8% (severe) vs. 66.4% (mild) compliance remaining at 96 h	Pass
7	Regional stress amplification	Gattinoni et al. [15]	~2-fold; acceptable range 1.5–2.5	Median peak amplification 1.60-fold	Pass
8	Plateau-pressure protocol boundary	ARDSNet practice [1]	Near 30–33 cm H_2_O safety boundary	Median severe-phenotype peak 33.0 cm H_2_O before volume reduction	Pass (boundary)
9	Maximum structural-compliance decline rate	Nieman et al. [20]	≤1.55% of baseline per hour	1.55%/h; boundary was active	Pass (boundary)

**Table 3:** Experiment 1. Ventilation strategies on matched virtual patients (*N* = 60, 96 h). Loss is structural (tissue) compliance.

Arm	Mean PEEP	Median Plateau	Structural Loss	vs. Practice
PEEP 5 (highly averse)	5.0	81.8	68.8%	+7.4
PEEP 8 (LUNG SAFE typical)	8.0	36.9	61.4%	—
PEEP 10	10.0	31.6	46.5%	−14.9
PEEP 15 (reached by necessity)	15.0	29.2	39.3%	−22.1
Aeration-targeted (aggressive)	20.2	33.5	52.7%	−8.7
Dose-minimizing, aggregated (legacy)	18.2	31.3	43.2%	−18.2
FAAST, compartmental (corrected)	13.5	29.9	40.7%	−20.7

**Table 4:** Experiment 2. Identical ventilation across phenotypes defined by their whole-lung compliance stratum. Global columns are the foil: what the clinician sees. Compartmental columns are the endpoints. Abbreviations: $C_{rs}$, respiratory-system compliance; ΔP, driving pressure; $r_{\sigma }$, suprathreshold stress ratio, calculated as effective regional stress divided by the model reference stress threshold; SASH, Strain-Accumulated Strain History; CDI, Continuum of Damage Index.

Phenotype	Crs	Plateau	ΔP	Clinician Response	rσ (Worst)	SASH (Worst)	CDI (Worst)
Healthy	87	14.8	4.8	would not act	0.81	10.2	0.012
L (mild)	43	19.8	9.8	would not act	1.40	19.5	0.062
I (intermediate)	32	23.0	13.0	would not act	1.58	27.6	0.117
H (severe)	21	30.1	20.1	would act	1.82	42.9	0.205

**Table 5:** Experiment 3. Out-of-sample evaluation of PEEP-selection strategies in a fresh virtual cohort (*N* = 50, independent seed, 96 h).

Strategy	Median Structural Loss	vs. Practice
PEEP 5	68.9%	+6.9
PEEP 8 (real-world practice)	62.0%	—
PEEP 12	41.2%	−20.8
PEEP 15	39.4%	−22.6
FAAST crossover	42.5%	−19.6
Compliance-maximizing (edge case)	59.2%	−2.8
Retrospective model-defined optimum	38.9%	−23.1

**Table 6:** Experiment 4. Mechanism-isolated run. The safety rail was disabled to preserve the assigned tidal-volume contrast in all four cells. Values report median structural-compliance loss at 96 h.

	VT 450 mL	VT 390 mL (Dead-Space Floor)	Dose Effect
PEEP 8 (practice)	63.1%	61.8%	1.2 points
FAAST crossover	43.5%	37.5%	5.9 points

**Table 7:** Experiment 4 compartmental endpoints in the protocol-realistic run. CDI > 0.5 is a prespecified model-internal severity cutoff corresponding to more than 55.6% local structural-compliance loss; it is not a validated clinical threshold.

Strategy	rσ (Worst)	CDI (Worst)	Mean CDI	Compartments with CDI > 0.5
PEEP 8 + VT 450 (practice)	1.49	0.913	0.762	17/20
PEEP 8 + VT 390	1.49	0.913	0.761	17/20
FAAST + VT 450	1.81	0.909	0.441	9/20
FAAST + VT 390 (both levers)	1.78	0.908	0.396	8/20

Abbreviations: VT, tidal volume; $r_{\sigma }$, suprathreshold stress ratio, calculated as effective regional stress divided by the model reference stress threshold; CDI, Continuum of Damage Index.

## Data Availability

No patient, animal, or new experimental datasets were generated in this computational study. The complete deterministic model implementation, experiment scripts, correspondence-fence code, model specification, and documentation supporting the reported in silico analyses are openly available in Zenodo, version 1.0.0, [19] at https://doi.org/10.5281/zenodo.21577653. The reported numerical results can be reproduced using the deposited code, documented model parameters, and specified random seeds. The software is investigational and is not intended for clinical use. Access and reuse are governed by the rights notice included in the archived repository.

## Supplementary Materials

The following supporting information can be downloaded at: https://www.mdpi.com/article/10.3390/jcm15197545/s1, Supplementary Methods and Sensitivity Analyses, including the complete compartmental equations, parameter and provenance register, detailed ventilator protocols, correspondence and sensitivity analyses, and reproducibility map. Supplementary Methods and Sensitivity Analyses; Table S1: Controller-blind correspondence fence for the phenotype-conditioned lung model; Table S2: Experimental roadmap: each experiment addresses one clinically framed model question; Table S3: Matched 2 × 2 factorial structure for Experiment 4; Table S4: Compartmental mechanics and threshold parameters; Table S5: Regional exposure, tissue-state, and recovery parameters; Table S6: Ventilator protocol and controller parameters; Table S7: One-at-a-time sensitivity of provisional barrier and RACE-pathway parameters: mild and severe columns report percentage of structural compliance remaining at 96 h under PEEP 8, whereas other strategy columns report median percentage structural-compliance loss at 96 h; Table S8: Operational-threshold sensitivity of the compartmental crossover: loss is modeled structural-compliance loss at 96 h; Table S9: Experiment 1 strain-history susceptibility-modifier ablation: loss is median modeled structural-compliance loss at 96 h; Table S10: Public reproducibility files and their roles.
