# Supplementary material for: Global Protective Ventilator Settings Are Not Regional Protection: A Compartment-Resolved Framework for Personalizing Mechanical Ventilation in Acute Respiratory Distress Syndrome
Source: J Clin Med. 2026 Sep 28;15(19):7545. doi: 10.3390/jcm15197545 (PMC13666132; doi:10.3390/jcm15197545)
Supplement: Supplementary file 1 [file jcm-15-07545-s001.zip › jcm-4478530-supplementary.pdf]

Supplementary Methods and Sensitivity Analyses

Global Protective Ventilator Settings Are Not Regional Protection

Michael S. Champagne, MSHSA, RRT, RPFT | Manuscript jcm-4478530 | 10 September 2026

Purpose. This supplement provides the mathematical and computational specification supporting the main article. It separates the phenotype-conditioned twenty-compartment test lung from the FAAST analytical engine, documents the controller-blind correspondence fence and model freeze, and supplies the protocol, parameter, sensitivity, and reproducibility details required to audit the reported in silico experiments. The supplement does not introduce a second model or alter any reported result. All outputs remain deterministic, model-conditional predictions rather than patient outcomes.

Contents

Section	Content
S1	Developmental sequence and evidentiary boundary
S2	Compartmental lung architecture and per-breath mechanics
S3	FAAST regional dose and tissue-state architecture
S4	Ventilator protocols and experiment specifications
S5	Parameter and provenance register
S6	One-at-a-time model-parameter sensitivity
S7	Crossover operational-threshold sensitivity
S8	Strain-history modifier ablation
S9	Reproducibility and execution map

S1 Developmental Sequence and Evidentiary Boundary

S1.1 Distinct Roles of the Analytical Engine and Test Lung

The study combined two computational components that serve different purposes. The Fatigue-Aware Alveolar Stress and Strain Tracking (FAAST) engine translates a complete ventilator prescription into regional estimates of mechanical participation, repeated alveolar collapse and expansion (RACE), overdistension, stress, strain, cumulative exposure, and modeled tissue state. The phenotype-conditioned test lung supplies the regional mechanical state on which that analysis operates. It contains twenty reduced-order compartments with declared differences in compliance, opening pressure, recruitability, surfactant state, epithelial-barrier integrity, and prior modeled injury. The lung model is therefore the experimental substrate; FAAST is the analytical layer used to interrogate what a ventilator pattern does to that substrate.

Panwar and colleagues' LUNG SAFE compliance strata supplied the clinical frame for the ARDS severity distribution [1]. Park and colleagues' observations in patients receiving long-term ventilation for amyotrophic lateral sclerosis served separately as a conceptual homeostatic floor [2]. The latter observations were not treated as an ARDS phenotype or as patient-level validation; they constrained the model against the biologically unreasonable assumption that every nonzero mechanical exposure must progress inexorably to failure.

S1.2 Why a Reduced-Order Simulation Was Used

FAAST requires synchronized information about regional participation, RACE activity, local deformation, cumulative mechanical exposure, and evolving tissue state. No available patient dataset jointly provides these measurements across alternative ventilator strategies. A deterministic reduced-order simulation was therefore used as a developmental test bench. It allowed the same starting lung to receive different prescriptions and the same prescription to be applied to different phenotypes, isolating model behavior without claiming patient-specific reconstruction. The intended next step is prospective comparison with patient waveforms, imaging, gas exchange, hemodynamics, biomarkers, and outcomes.

S1.3 Controller-Blind Correspondence and Freeze

Model construction preceded intervention testing. During the correspondence phase, only conventional ventilation was used; FAAST-guided management was excluded. No FAAST outcome was used to select the phenotype distribution, recruitability distribution, baseline mechanics, injury kinetics, or correspondence tolerances. After the nine prespecified gates were satisfied in 200 virtual lungs generated with seed 20260710, the phenotype substrate and its governing parameters were frozen before the strategy experiments began.

Correspondence is used deliberately rather than validation. Passing the fence means that the reduced-order model occupies a clinically recognizable mechanical domain and reproduces the selected direction and temporal behavior of conventionally ventilated acute lung injury. It does not establish patient-specific predictive accuracy, clinical efficacy, or correspondence of the Continuum of Damage Index (CDI) to a clinical endpoint.

#	Target	External anchor	Tolerance	Model	Result
1	Phenotype distribution (mild/intermediate/severe)	Panwar and LUNG SAFE [1]	12%/14%/74%, sampling tolerance	11.5%/15.0%/73.5%	Pass
2	Recruitability distribution (mean/median)	Gattinoni et al. [3]	Mean 0.13; median 0.09	Mean 0.116; median 0.091	Pass
3	Severe-phenotype baseline mechanics	LUNG SAFE and Amato et al. [1,4]	Compliance 20-30 mL/cmH2O; ΔP 15-18 cmH2O	27.4 mL/cmH2O; 16.4 cmH2O	Pass
4	Onset of modeled injury acceleration	Caironi and Gattinoni et al. [5]	18 ± 11 h	20.0 h	Pass
5	Injury-trajectory form	Yamaguchi et al. [6]	Lag, acceleration, bounded plateau	Satisfied; peak decline at 20.6 h	Pass
6	Severity ordering	Clinical acute-lung-injury behavior	Greater deterioration, severe > mild	56.8% (severe) vs 66.4% (mild) compliance remaining at 96 h	Pass
7	Regional stress amplification	Gattinoni et al. [7]	~2-fold; acceptable range 1.5-2.5	Median peak amplification 1.60-fold	Pass
8	Plateau-pressure protocol boundary	ARDSNet practice [8]	Near 30-33 cmH2O safety boundary	Median severe-phenotype peak 33.0 cmH2O before volume reduction	Pass (boundary)
9	Maximum structural-compliance decline rate	Nieman et al. [9]	≤ 1.55% of baseline per hour	1.55%/h; boundary was active	Pass (boundary)

Table S1. Controller-blind correspondence fence for the phenotype-conditioned lung model.

Two construction changes were made before the freeze to improve correspondence with external observations. Recruitability was changed from a symmetric to a right-skewed distribution to reproduce the reported mean-to-median relationship, and the rate of structural-compliance decline was bounded by the steepest selected experimental rate. A later code review corrected a pathway-gating error so that epithelial-barrier state modifies RACE-mediated atelectrauma while overdistension can act on an intact compartment. The same nine gates were rerun after that correction and remained satisfied without changing their definitions or phenotype distributions.

The compartmental crossover was developed after the legacy summed-dose controller failed to preserve opposing regional states. The frozen lung substrate was not retuned when the crossover was introduced. Its post hoc origin and threshold dependence are retained explicitly; it is evaluated as a testable decision hypothesis rather than an independently validated optimum.

S2 Compartmental Lung Architecture and Per-Breath Mechanics

Model architecture. Each virtual lung contained twenty parallel respiratory compartments arranged from nondependent to dependent along a gravitational axis. The compartments shared the same applied airway pressure but differed in structural compliance, superimposed-pressure offset, opening pressure, recruitability, epithelial-barrier integrity, surfactant state, fatigue history, and prior modeled injury. Each compartment should be understood as a reduced-order mechanical element rather than a discrete anatomical alveolus. This architecture increases the resolution of the model from a single whole-lung value to twenty state-bearing elements, allowing the same ventilator breath to interact differently with tissue that is open, unstable, poorly recruitable, or already carrying disproportionate load.

Antecedent ARDS severity entered the model through this initial mechanical architecture. Greater severity reduced functional residual capacity, increased the gravitational and opening-pressure gradients, lowered initial barrier integrity in dependent regions, and increased the proportion of poorly recruitable tissue. Native lung injury was therefore represented as the condition of the lung at the start of ventilation rather than as a separately imposed inflammatory process. This was a deliberate boundary of the reduced-order model. It allowed the experiments to examine how ventilation acts upon an already injured lung without assigning unsupported kinetics to inflammation, edema formation, or biological progression independent of the modeled mechanical pathways.

The phenotype-conditioned lungs were positioned within clinically recognizable compliance ranges using static respiratory-system compliance:

$$C_{\mathrm{rs}} = \frac{V_T}{P_{\mathrm{plat}} - \mathrm{PEEP}} = \frac{V_T}{\Delta P}$$

where V_T is tidal volume, P_{plat} is plateau pressure, PEEP is positive end-expiratory pressure, and ΔP is driving pressure. This whole-lung value provided the clinical anchor for the phenotype bands described in Supplementary Section S1. It did not determine how the breath was distributed within the lung. That distribution emerged from the state of the individual compartments.

Recruitment state. Compartmental openness was represented as a continuous sigmoid function of the pressure available to that compartment relative to its opening pressure:

$$a_i(P) = \frac{1}{1 + \exp\left[-\left(P - P_{\mathrm{sup},i} - P_{\mathrm{open},i}\right)/w_o\right]}$$

where a_i ranges from 0 for a functionally closed compartment to 1 for a fully open compartment, $P_{\mathrm{sup},i}$ is the regional superimposed-pressure offset, $P_{\mathrm{open},i}$ is the compartmental opening pressure, and w_o controls the width of the opening transition. Openness was calculated at end expiration using PEEP and again at end inspiration using plateau pressure. The sigmoid formulation avoids treating recruitment as an instantaneous all-or-none event and permits partially open or unstable compartments near their opening pressure.

Surfactant impairment increased the opening pressure according to

$$P_{\mathrm{open},i}(t) = P_{\mathrm{open},0,i}\left[1 + \gamma_{\mathrm{surf}} S_{\mathrm{surf},i}(t)\right]$$

where $P_{\mathrm{open},0,i}$ is the initial opening pressure, $S_{\mathrm{surf},i}$ is the modeled surfactant-deactivation state, and γ_{surf} determines its influence. This creates a mechanically plausible feedback: an unstable compartment may become more difficult to reopen as surfactant function deteriorates, while a stable compartment may retain or recover mechanical availability.

Driving pressure and tidal-volume distribution. The pressure required to deliver the prescribed tidal volume depended on the compliance that remained available at end expiration:

$$\Delta P = \frac{V_T}{\sum_{i=1}^{20} a_{\mathrm{EE},i}\, C_{\mathrm{struct},i}}$$
$$P_{\mathrm{plat}} = \mathrm{PEEP} + \Delta P$$

where $a_{\mathrm{EE},i}$ is end-expiratory openness and $C_{\mathrm{struct},i}$ is the current structural compliance of compartment i. Tidal volume was then distributed in proportion to each compartment's cycle-mean openness and structural compliance:

$$\Delta V_i = V_T \frac{\bar{a}_i C_{\mathrm{struct},i}}{\sum_{j=1}^{20} \bar{a}_j\, C_{\mathrm{struct},j}},\ \bar{a}_i = \frac{a_{\mathrm{EE},i} + a_{\mathrm{EI},i}}{2}$$

where $a_{\mathrm{EI},i}$ is end-inspiratory openness. This allocation is the model's central load-shifting mechanism. Closed and refractory compartments accept little of the delivered breath. Compartments that remain open and compliant therefore accept a larger share, even when the prescribed tidal volume and measured airway pressures remain within familiar lung-protective limits. As functional lung volume decreases, the same global breath is concentrated into progressively fewer available units.

Regional strain. Strain was calculated from regional pressure and volume change using a cube-root specific-elastance formulation [10]. The regional pressure available at end expiration was

$$\Delta P_{L,\mathrm{PEEP},i} = \max\left(\mathrm{PEEP} - P_{\mathrm{sup},i}, 0\right)$$

and the static and peak linear strains were

$$\varepsilon_{\mathrm{static},i} = \left(1 + \frac{\Delta P_{\mathrm{L,PEEP},i}}{E_{\mathrm{spec}}}\right)^{1/3} - 1$$

$$\varepsilon_i = \left(1 + \frac{\Delta P_{\mathrm{L,PEEP},i}}{E_{\mathrm{spec}}} + \frac{\Delta V_i}{V_{\mathrm{ref},i}}\right)^{1/3} - 1$$

where E_{spec} is specific elastance and $V_{\mathrm{ref},i}$ is the compartmental reference volume after accounting for recruitment. The cube-root transformation converts volumetric expansion to a linear deformation measure. The tidal strain excursion was $\Delta\varepsilon_i = \varepsilon_i - \varepsilon_{\mathrm{static},i}$, and its rate was approximated as $\dot{\varepsilon}_i = \Delta\varepsilon_i / T_i$, where T_i is inspiratory time.

Effective regional stress. Effective wall stress was represented as the sum of three contributions:

$$\sigma_{\mathrm{eff},i} = K_E \varepsilon_i + (K_{\mathrm{local}} - 1) K_E \varepsilon_i |\nabla a_i| + \kappa_T K_E \dot{\varepsilon}_i$$

The first term represents equilibrium stress associated with regional deformation. The second represents stress concentration where adjacent compartments differ in openness. This term approaches zero in a mechanically uniform lung and increases at interfaces between open and poorly open regions. The third represents a rate-dependent viscoelastic transient. Although the local concentration coefficient was bounded by a sixfold ceiling derived from experimental and computational junction behavior, the regional amplification that emerged in the correspondence analysis was approximately 1.6-fold, within the range reported for heterogeneous injured

lung tissue[9,12]. The model was therefore permitted to express stress amplification when heterogeneity was present but was not forced to produce a predetermined regional stress raiser.

Operational representation of RACE. Repeated alveolar collapse and expansion was represented by the change in compartmental openness across the breath, weighted by the degree to which the compartment remained closed at end expiration:

$$R_{\mathrm{RACE},i} = \max\left[\left(a_{\mathrm{EI},i} - a_{\mathrm{EE},i}\right)\left(1 - a_{\mathrm{EE},i}\right), 0\right]$$

A compartment that remained open throughout the breath had little or no RACE activity. A compartment that opened during inspiration but returned toward closure at end expiration produced a larger value. This is a reduced-order representation of RACE rather than direct visualization of individual alveoli. It translates the clinically recognized process of repeated opening and closing into a compartmental quantity that can be followed across breaths. In the crossover experiment, a compartment was counted as RACE-active when this value exceeded the prespecified operational cutoff; the sensitivity of PEEP selection to that cutoff was examined separately.

Thresholds and implementation boundary. The operative mechanical threshold in this experimental implementation was a linear strain of

$$\varepsilon_{\mathrm{th}} = 0.30$$

The corresponding stress-domain reference was derived through the model's scale-bridging modulus:

$$\sigma_{\mathrm{th}} = K_E \varepsilon_{\mathrm{th}} \approx 49.8\ \mathrm{kPa}$$

The suprathreshold stress ratio was therefore

$$r_{\sigma,i} = \frac{\sigma_{\mathrm{eff},i}}{\sigma_{\mathrm{th}}}$$

with $r_{\sigma,i} > 1$ identifying a compartment that had entered the model's suprathreshold loading regime. The value of σ_{th} is a model-consistent reference scale, not an independently measured universal alveolar rupture stress. Likewise, crossing either threshold indicates entry into a modeled injury regime rather than a diagnosis of histological injury.

The companion theoretical paper develops the broader FAAST stress–strain framework and uses a volumetric strain convention [13]. The deposited implementation used to generate the present experiments predates that refinement and calculates linear strain through the cube-root formulation shown above. All strain thresholds, stress ratios, and reported outcomes in this manuscript refer to this experimental implementation. This distinction preserves reproducibility without implying that the reduced-order model resolves the full tissue-level stress field. Complete parameter values, provenance classifications, and executable equations are provided in Section S5 and public code archive.

S3 FAAST Regional Dose and Tissue-State Architecture

The per-breath calculations described above determine where each breath is delivered and what regional stress, strain, and RACE activity it produces. FAAST adds the longitudinal layer: it tracks how those regional exposures accumulate and how the modeled tissue state changes over time. The framework therefore separates four related but noninterchangeable quantities: the ventilator prescription, the mechanical state of the lung, the regional burden delivered by each breath, and the modeled tissue response to that burden.

The general FAAST framework is intended to analyze the complete ventilator prescription rather than PEEP in isolation. In the present prototype, PEEP, tidal volume, respiratory rate, and inspiratory time entered the mechanical calculations. PEEP and tidal volume were varied in the intervention experiments, while respiratory rate and inspiratory time were held at their stated experimental values. Detailed inspiratory-flow and ventilator-waveform behavior was not simulated in this build. The model should therefore be understood as a reduced-order test of regional dose allocation and management logic rather than a complete representation of ventilator or respiratory-system dynamics.

Regional exposure summaries. For each compartment, mean inspiratory stress was approximated from the static and peak effective stresses:

$$\bar{\sigma}_i = \frac{\sigma_{\mathrm{static},i} + \sigma_{\mathrm{eff},i}}{2}$$

Tonic alveolar stress per breath was then estimated as

$$\mathrm{TAS}_{b,i} = \bar{\sigma}_i T_i$$

where T_i is inspiratory time. Time-Accumulated Stress History (TASH) accumulates this stress-time exposure across breaths:

$$\mathrm{TASH}_i(t) = \frac{1}{3600} \sum_{b=1}^{N_b(t)} \mathrm{TAS}_{b,i}$$

TASH therefore increases when a compartment bears greater wall stress, remains stressed for a larger fraction of each breath, or is exposed to more breaths over time. It is sensitive to sustained distending load and to the tonic component of PEEP-related stress, but it remains a regional exposure measure rather than a direct measure of tissue injury.

Cyclic strain-energy exposure per breath was approximated as

$$\mathrm{SAS}_{b,i} = \bar{\sigma}_i \Delta\varepsilon_i$$

where $\Delta\varepsilon_i$ is the tidal strain excursion. Strain-Accumulated Strain History (SASH) accumulates this cyclic deformation burden:

$$\mathrm{SASH}_i(t) = \frac{1}{3600} \sum_{b=1}^{N_b(t)} \mathrm{SAS}_{b,i}$$

The factor $1/3600$ converts the stress–time accumulation used for TASH from seconds to hours. In the SASH calculation, the same factor is retained as an inherited numerical reporting scale and does not convert stress–strain work into kPa·h. SASH is therefore reported in model-scaled units in this implementation. The companion theoretical formulation retains the same strain-accumulated stress concept but formalizes it as the cumulative positive loading-branch integral $\sum \int \sigma \, d\varepsilon$, with units of kPa, equivalent to energy per unit reference volume. Numerical SASH values from the two implementations should not be compared directly.

SASH increases when a compartment repeatedly absorbs deformation work. It is therefore sensitive to tidal-volume concentration, regional compliance, recruitment state, and the number of delivered breaths. A stiff compartment may carry high stress with limited deformation, whereas a compliant or unstable compartment may absorb substantial deformation under the same airway pressure. TASH and SASH preserve this distinction between sustained load and cyclic deformation.

These quantities are reduced-order indices reported on the common accumulation scale used by the deposited implementation. They support comparisons among compartments, phenotypes, and ventilator strategies within the model; they should not be interpreted as directly measured absolute tissue energies. Importantly, TASH and SASH are not added together and then inserted into the Continuum of Damage Index (CDI). They describe what mechanical burden was delivered. The active injury-state calculation uses a separate normalized and threshold-sensitive kernel so that the same exposure is not counted again merely because it has also been reported as TASH or SASH.

Direct strain and stress pathway. The model first normalizes regional strain and effective stress to their respective reference thresholds:

$$r_{\varepsilon,i} = \frac{\varepsilon_i}{\varepsilon_{\text{th}}}, \; r_{\sigma,i} = \frac{\sigma_{\text{eff},i}}{\sigma_{\text{th}}}$$

A smooth suprathreshold operator was used to avoid a discontinuous transition at either threshold:

$$g(x) = \frac{\ln\left(1 + e^{k_s x}\right)}{k_s}$$

The combined suprathreshold mechanical term was

$$S_i = w_\varepsilon\left[g\left(r_{\varepsilon,i} - 1\right)\right]^2 + w_\sigma\left[g\left(r_{\sigma,i} - 1\right)\right]^2$$

where w_ε and w_σ weight the strain and stress contributions. The squared response causes the modeled burden to accelerate as regional exposure moves farther above the reference boundary while preserving a smooth transition near that boundary.

The direct strain and stress pathway was then expressed as

$$\psi_{\text{V},i} = \psi_{\text{floor}} + \lambda S_i (1 + \kappa_F F_i)$$

where ψ_{floor} supplies a small nonzero background exposure, λ scales the suprathreshold term, and F_i is a cumulative strain-history state:

$$\frac{dF_i}{dt} = \left[\max\left(r_{\varepsilon,i} - 1,0\right)\right]^{q_F}$$

This state allows prior suprathreshold strain to modify susceptibility to the current mechanical insult. It is a provisional fatigue modifier rather than a third injury pathway or a separately reported bedside index. Its

influence was therefore examined by ablation during sensitivity analysis. The principal comparison with practice PEEP remained directionally unchanged when the modifier was removed, although the relative ordering of the two dose-guided controllers was sensitive to this assumption.

RACE-mediated pathway. Repeated alveolar collapse and expansion entered through a separate atelectrauma pathway:

$$\psi_{\mathrm{RACE},i} = \kappa_{\mathrm{RD}} R_{\mathrm{RACE},i}(1 + A_{\mathrm{RD}})$$

where $R_{\mathrm{RACE},i}$ is the compartmental opening-and-closing activity defined in Supplementary Section S2, κ_{RD} scales its contribution, and A_{RD} represents amplification after repetitive epithelial disruption.

The model does not assume that every opening-and-closing event immediately produces equivalent structural injury. A cumulative RACE state, $N_{\mathrm{RD},i}$, tracks repeated instability while allowing recovery during mechanically stable periods:

$$\frac{dN_{\mathrm{RD},i}}{dt} = R_{\mathrm{RACE},i}\frac{\mathrm{RR}}{\mathrm{RR}_{\mathrm{ref}}} + \alpha_{\mathrm{spread}}(1 - \bar{B}) - \rho_{\mathrm{RD}}\left(1 - R_{\mathrm{RACE},i}\right)$$

where RR is respiratory rate, $\mathrm{RR}_{\mathrm{ref}}$ is the reference rate, $\bar{B}$ is mean barrier integrity across the modeled lung, α_{spread} is a provisional mean-field injury-spread term, and ρ_{RD} represents recovery when the compartment remains stable. The associated epithelial-barrier state was

$$B_i = \frac{1}{1 + \exp\left[k_B\left(N_{\mathrm{RD},i} - N_{\mathrm{crit},i}\right)\right]}$$

where B_i approaches 1 when barrier integrity is preserved and falls after the cumulative RACE state passes the compartment-specific critical region. Dependent compartments were assigned lower critical values, reflecting their greater initial vulnerability. This formulation produces an early period during which the barrier tolerates repeated instability, followed by more rapid loss after the modeled resilience boundary is exceeded. It is the reduced-order counterpart of the lag followed by abrupt barrier deterioration observed in experimental epithelial systems [6].

The two injury pathways were intentionally gated differently:

$$\psi_{\mathrm{eff},i} = \psi_{\mathrm{V},i} + (1 - B_i)\psi_{\mathrm{RACE},i}$$

Suprathreshold strain and stress act directly because an overdistended compartment does not require prior epithelial-barrier failure to be injured by stretch. In contrast, the structural consequence assigned to RACE increases as barrier integrity is lost. An earlier implementation incorrectly applied the barrier gate to both pathways, which suppressed overdistension injury in compartments with an intact barrier. The corrected formulation restricts the barrier gate to the RACE-mediated pathway. As reported in Supplementary Section S1, the controller-blind correspondence fence continued to pass all nine gates after this correction without retuning the phenotype substrate.

Continuum of Damage Index. The effective pathway input advances the compartmental CDI according to

$$\frac{d\,\mathrm{CDI}_i}{dt} = (1 - \mathrm{CDI}_i)\psi_{\mathrm{eff},i} - \rho_C \mathrm{CDI}_i$$

where ρ_C is the modeled recovery term. CDI is restricted to the interval from 0 to 1. The factor $1 - \mathrm{CDI}_i$ progressively reduces further accumulation as CDI approaches its upper boundary, producing a bounded rather than indefinitely increasing state.

A slower structural-memory state prevents an immediate return of compliance when the current injury signal decreases:

$$\mathrm{CDI}_{\mathrm{mem},i}(t+\Delta t) = \max\left[\mathrm{CDI}_i(t+\Delta t), \mathrm{CDI}_{\mathrm{mem},i}(t) - \lambda_{\mathrm{mem}}\Delta t\right]$$

Structural compliance was then mapped from that retained state:

$$C_{\mathrm{struct},i}(t) = \frac{C_{0,i}}{1 + \delta\,\mathrm{CDI}_{\mathrm{mem},i}(t)}$$

with $\delta = 2.5$. At the upper CDI boundary, this mapping approaches 28.6% of baseline structural compliance. The rate at which compliance could decline was additionally limited to the experimentally anchored ceiling described in the correspondence fence.

The prespecified reporting cutoff of $\mathrm{CDI} > 0.5$ is entirely model internal. Under the stated mapping,

$$\frac{C_{\mathrm{struct}}}{C_0} = \frac{1}{1 + 2.5(0.5)} = 0.444$$

so the cutoff corresponds algebraically to more than 55.6% modeled loss of local structural compliance. It has no established correspondence to histological injury, oxygenation, biomarker concentration, mortality, or a clinical diagnosis. Compartments above this boundary should therefore be described as having crossed the model's internal high-damage threshold, not as clinically or functionally lost lung tissue.

This separation between exposure and consequence is central to interpreting the results. A decrease in the subsequent rate of TASH or SASH accumulation does not necessarily indicate recovery. It may mean that a compartment has stabilized and is receiving a more tolerable breath, but it may also mean that the compartment has stiffened, closed, or ceased to participate mechanically. Conversely, a compartment may accumulate greater exposure because recruitment has restored its participation while its modeled tissue state remains comparatively preserved. FAAST retains both layers so that the mechanical economy of a ventilator adjustment is not judged solely by the amount of lung recruited or by the immediate reduction of a single global variable.

Validity boundary. Respiratory rate enters this implementation through the RACE event counter relative to a reference rate of 15 breaths/min. It does not independently scale the direct strain and stress pathway. The present build is therefore appropriate only for respiratory rates near the calibrated range and was not used to infer tidal-volume–respiratory-rate tradeoffs at fixed minute ventilation. The parameter values, provenance classifications, state-update equations, and sensitivity ranges are provided in Section S5 and executable public model specification.

S4 Ventilator Protocols and Experiment Specifications

The experiments were designed to examine the mechanical economy of the breath from four related perspectives. PEEP determined how much potentially recruitable lung remained available at end expiration, while tidal volume determined the dose distributed among the compartments that participated in the next breath. Respiratory rate and inspiratory time influenced exposure duration and RACE event density but were held constant within each experiment. This allowed the study to isolate defined questions without implying that any single ventilator setting operates independently of the others.

The four experiments followed a common progression. Experiment 1 compared alternative recruitment and regional-analysis strategies. Experiment 2 asked whether the same globally protective breath produced different regional consequences when applied to different lung phenotypes. Experiment 3 evaluated whether a compartmental count could identify the transition between unresolved RACE and increasing overdistension as PEEP was raised. Experiment 4 then tested whether recruitment changed the mechanical effect of reducing tidal volume.

Experiment	Question	Design	Primary model output
1	Does aggregation change strategy ranking?	N=60 matched; seven strategies; 96 h	PEEP and structural-compliance loss
2	Can acceptable global settings conceal regional burden?	Four prescribed phenotypes; fixed ventilation; 24 h	Worst regional stress ratio, SASH, and CDI
3	Can a compartment count locate a PEEP tradeoff?	PEEP 4-24; fresh N=50 cohort	Selected PEEP and loss versus practice and model optimum
4	Does recruitment modify the tidal-volume effect?	Matched N=50, 2 × 2 factorial	Paired effects and interaction

Table S2. Experimental roadmap. Each experiment addresses one clinically framed model question.

S4.1 Common Ventilator Protocol and Safety Envelope

Simulations used an idealized reference patient with a predicted body weight of 70 kg. Experiments 1, 3, and 4 began with a tidal volume of 450 mL, respiratory rate of 15 breaths/min, and inspiratory time of 1.0 s. The 450-mL tidal volume corresponds to approximately 6.4 mL/kg and was selected as a clinically familiar order within the lung-protective range rather than as an assertion that 450 mL is preferable to the exact 6 mL/kg value of 420 mL. Experiment 2 used 420 mL, or 6 mL/kg, with a respiratory rate of 18 breaths/min and an inspiratory time of 1.0 s because its purpose was to compare regional behavior under an identical conventionally protective breath.

The canonical simulation horizon was 96 h. Experiment 2 used a 24-h horizon because it examined early divergence between global and regional measurements rather than long-term strategy performance. State variables were updated at intervals of 0.1 h. Each interval used a representative breath, with cumulative exposure and RACE activity scaled to the number of breaths delivered during that interval.

All clinically realistic strategy comparisons incorporated an ARDSNet-style pressure safety rail [8]. When plateau pressure exceeded 33 cmH_2O, tidal volume was reduced in steps to a lower boundary of 390 mL in the 450-mL protocol. This trigger was intentionally set above the conventional 30-cmH_2O plateau target to allow a 10% tolerance before automatic reduction. Experiment 2 used the corresponding proportional lower boundary because its starting tidal volume was 420 mL.

The tidal-volume floor was a model constraint, not a simulated gas-exchange target. The model does not calculate carbon-dioxide production, physiological dead space, pH, or the respiratory rate required to sustain alveolar ventilation at progressively smaller tidal volumes. It therefore cannot determine whether a lower tidal volume would provide adequate ventilation in an individual patient. The floor prevented the experimental protocol from continuing to reduce tidal volume into a range where dead-space ventilation would be expected to dominate.

FAAST is evaluated here as an analytical layer operating within a clinically viable ventilator envelope. Any future clinical recommendation would remain subordinate to oxygenation, carbon-dioxide clearance, acid–base status, airway-pressure limits, hemodynamic and right-ventricular tolerance, patient–ventilator interaction, and

clinician judgment. Evidence of circulatory compromise would override a mechanically derived recommendation to increase or maintain PEEP.

S4.2 Ventilator Strategies and Comparators

Four fixed-PEEP arms were included in Experiment 1: 5, 8, 10, and 15 cmH_2O. PEEP of 8 cmH_2O approximated the central tendency reported for patients with ARDS in LUNG SAFE [1] and served as the principal practice comparator. PEEP of 5 cmH_2O represented a low-recruitment boundary, while 10 and 15 cmH_2O provided progressively greater fixed recruitment. These arms were not intended to represent complete clinical protocols. They isolated the effect of maintaining different end-expiratory lung states while the remaining ventilator settings and the starting lung were held constant.

An aggressive aeration-targeted comparator was included to provide a stronger conventional recruitment benchmark. This arm began at PEEP 8 cmH_2O and reassessed mean end-expiratory openness every 4 h. If mean openness remained below 0.65, PEEP was increased by 2 cmH_2O to a maximum of 22 cmH_2O. Because gas exchange was not modeled, this strategy did not calculate oxygenation and should not be interpreted as an oxygenation-response protocol. Rather, end-expiratory openness served as a reduced-order aeration surrogate for an aggressive clinical strategy that continues to raise PEEP while recruitment appears incomplete. Its inclusion prevented the FAAST comparison from depending solely on an inadequately recruited conventional control.

The legacy dose-guided controller was retained as a negative analytical result. Every 6 h, it searched candidate PEEP values from 6 to 24 cmH_2O and minimized the sum of the compartmental injury signals, with additional penalties for insufficient mean end-expiratory openness and PEEP above 18 cmH_2O. Although the mechanical quantities were calculated regionally, the objective collapsed them into a single sum. A few severely overdistended compartments could therefore be offset numerically by improvement in a larger number of less affected compartments. The controller reproduced the same loss of spatial information that FAAST was designed to expose.

The FAAST crossover rule was developed after this limitation became evident. Every 6 h, candidate PEEP values from 6 to 24 cmH_2O were evaluated without summing regional injury into a single objective. At each PEEP, the model counted compartments with RACE activity above the operational threshold and compartments with effective stress above the reference boundary:

$$n_{\mathrm{RACE}} = \sum_i I\left(R_{\mathrm{RACE},i} > 0.05\right)$$

$$n_{\mathrm{over}} = \sum_i I\left(r_{\sigma,i} > 1\right)$$

where $I(\cdot)$ is an indicator function. The selected PEEP was the lowest candidate value at which

$$n_{\mathrm{over}} \geq n_{\mathrm{RACE}}$$

The rule therefore locates a regional crossover rather than minimizing a global dose score. It does not use fitted weights to declare one RACE-active compartment equivalent to a specified amount of overdistension. It does, however, depend on the operational definitions of RACE and suprathreshold stress. Its post hoc origin and threshold dependence are therefore disclosed explicitly, and both thresholds were examined in the sensitivity

analysis. The crossover is evaluated as a testable decision hypothesis, not as a validated patient-specific PEEP target.

The archived code retains the legacy label “aggressive (oxygenation)” for this arm; because gas exchange was not modeled, the manuscript refers to it as aggressive aeration-targeted recruitment.

S4.3 Experiment 1 Recruitment and Strategy Comparison

Experiment 1 used 60 virtual lungs sampled once from the frozen phenotype and recruitability distributions with seed 20260710. Each virtual lung was copied into all seven intervention arms so that every strategy began with the same severity, recruitability, regional opening pressures, barrier state, and structural compliance. Each copy was then ventilated independently for 96 h. Differences among arms therefore arose from the applied strategy rather than from differences in the starting lungs.

The seven arms were fixed PEEP at 5, 8, 10, and 15 cmH_2O; aggressive aeration-targeted recruitment; the legacy summed-dose controller; and the FAAST compartmental crossover. The pressure safety rail was active in every arm. The principal outcome was percentage loss of modeled structural compliance at 96 h:

$$\text{Structural loss} = 100\left(1 - \frac{C_{\text{struct}}(96\text{ h})}{C_{\text{struct}}(0)}\right)$$

Mean PEEP, median driving pressure, median plateau pressure, phenotype-specific results, and the direction of each matched comparison were also reported. The experiment was designed first to determine how strongly recruitment altered modeled injury and then to determine whether preserving or aggregating regional information changed the behavior of a dose-guided strategy.

S4.4 Experiment 2 Regional Observability Under Identical Ventilation

Experiment 2 examined the information available to the clinician when the ventilator remained unchanged but the condition of the lung differed. Four prescribed mechanical states were used: a preserved-mechanics reference and mild, intermediate, and severe injured-lung phenotypes. Their baseline respiratory-system compliances were set to approximately 90, 45, 35, and 25 mL/cmH_2O, respectively. Increasing severity progressively reduced the number of readily available compartments and increased the number of intermediate or refractory compartments.

Every phenotype received identical ventilation: tidal volume 420 mL, PEEP 10 cmH_2O, respiratory rate 18 breaths/min, and inspiratory time 1.0 s. PEEP was deliberately held fixed. Allowing recruitment to change between phenotypes would have altered both the lung and the intervention, obscuring the question this experiment was intended to answer.

The global observations were respiratory-system compliance, plateau pressure, and driving pressure. Regional outputs included the fraction of tidal volume accepted by the most mechanically available compartments, effective stress, the worst-compartment stress ratio, SASH, and CDI at 24 h. Plateau pressure of 28 cmH_2O and driving pressure of 15 cmH_2O defined a prespecified no-action band for descriptive comparison. This band was not treated as a biological safety threshold. It represented a clinically familiar range in which the global mechanics alone would not ordinarily compel an immediate ventilator change.

The experiment asked whether two statements could be true at the same time: the airway-derived measurements could remain reassuring, while a smaller mechanically available portion of the lung accepted a disproportionate share of the breath and entered a suprathreshold regional state. It was therefore an observability experiment rather than a comparison of treatment efficacy.

S4.5 Experiment 3 Recruitment–Overdistension Crossover

Experiment 3 examined the regional state across candidate PEEP values from 4 to 24 cmH_2O in 1-cmH_2O increments. At each step, the model recorded end-expiratory openness, the number of recruited compartments, the number of RACE-active compartments, the number of suprathreshold compartments, plateau pressure, driving pressure, respiratory-system compliance, and the maximum regional stress ratio.

The crossover rule was first examined in representative moderate and severe phenotypes and was then applied without changing the frozen lung model to a fresh cohort of 50 virtual lungs generated with seed 77777. This second seed reduced dependence on the cohort used during model construction and the initial strategy comparison, but it remained an internal, within-model evaluation rather than external validation.

For each virtual lung, the 96-h structural-compliance loss associated with the crossover-selected PEEP was compared with fixed PEEP values of 5, 8, 10, 12, 15, and 17 cmH_2O. Two model-defined reference strategies were also calculated. The first selected the PEEP producing the greatest respiratory-system compliance and served as an edge case illustrating the behavior of compliance maximization. The second retrospectively identified the PEEP producing the lowest 96-h modeled structural loss across the completed sweep. Because this retrospective optimum requires knowledge of future model outcomes, it is not a clinically available strategy and was used only as a benchmark.

The principal outputs were selected PEEP, modeled structural-compliance loss, paired-case direction relative to PEEP 8 cmH_2O, and proximity to the retrospective model-defined optimum. Sensitivity to the RACE and stress-ratio cutoffs was evaluated separately in Supplementary Section S7.

S4.6 Experiment 4 Interaction Between Recruitment and Tidal-Volume Reduction

Experiment 4 tested the clinical proposition that the effect of tidal-volume reduction may depend on how much lung has first been made mechanically available. Fifty virtual lungs generated with seed 20260710 were evaluated in a matched 2 × 2 factorial design. Each lung served as its own control and was run through all four cells:

PEEP assignment	Tidal volume 450 mL	Tidal volume 390 mL
PEEP 8 cmH_2O	Cell A	Cell B
FAAST crossover PEEP	Cell C	Cell D

Table S3. Matched 2 × 2 factorial structure for Experiment 4.

The recruitment effect was calculated within each virtual lung as $A - C$. The tidal-volume effect before recruitment was $A - B$, and the residual tidal-volume effect after crossover recruitment was $C - D$. The combined effect was $A - D$. The interaction was defined as the increase in tidal-volume effect after recruitment:

$$\text{Interaction} = (C - D) - (A - B)$$

which is algebraically equivalent to

$$(A - D) - [(A - C) + (A - B)]$$

A positive value indicates that tidal-volume reduction produced a larger modeled benefit after the regional recruitment state had changed.

The factorial experiment was run in two forms. The protocol-realistic analysis retained the plateau-pressure safety rail. This reflected how the assigned tidal volume could be reduced in response to an excessive plateau pressure, but it also allowed the two low-PEEP cells to converge if the rail reduced the 450-mL assignment toward the 390-mL floor. That analysis was retained because the convergence itself describes the behavior of a clinically constrained protocol.

A second mechanism-isolation analysis disabled the pressure safety rail so that tidal volume remained at its assigned value in every cell. This preserved the factorial contrast and allowed the recruitment–tidal-volume interaction to be examined. It was not treated as a clinically acceptable ventilation protocol because the low-PEEP, higher-tidal-volume cell could generate plateau pressures that a clinician would not accept. No clinical recommendation was derived from that rail-disabled condition.

Primary outcomes were paired differences in structural-compliance loss. Worst-compartment stress ratio, worst and mean CDI, and the number of compartments crossing the internal $\mathrm{CDI} > 0.5$ reporting boundary were secondary model outputs. These measures were included to determine whether an improvement in the whole-lung result reflected broad preservation of regional participation or merely redistribution of injury into fewer compartments.

S4.7 Descriptive Analysis

The mechanical simulations were deterministic after each virtual lung had been generated. The study therefore did not use p-values, confidence intervals, hazard ratios, or survival analysis as evidence of treatment effect. Results were summarized using medians, interquartile ranges where informative, paired within-lung differences, and the proportion of matched cases in which one strategy produced a lower modeled outcome than another.

These percentages describe consistency within the simulated cohort; they are not estimates of clinical response probability. The appropriate evidentiary level is model-conditional mechanical prediction. External patient waveforms, imaging, gas-exchange measurements, biomarkers, hemodynamic observations, and clinical outcomes will be required to determine whether the predicted regional states correspond to patient physiology and whether they can support prospective ventilator guidance.

S5 Parameter and Provenance Register

The tables below identify the fixed values used by the reported implementation. Literature anchor denotes a value or boundary taken from the cited physiological or clinical literature. Derived denotes an algebraic consequence of another declared value. Fixed modeling assumption denotes a value selected to complete the reduced-order mechanism without claiming that it is a universal tissue constant. Sensitivity tested identifies provisional quantities that were varied in the reported robustness analyses. The executable code remains the authority for exact implementation.

Parameter	Value	Role	Basis
N	20	Parallel regional compartments	Fixed architecture
C_UNIT	6.0 mL/cmH_2O	Baseline compliance scale per compartment	Clinical scale
V_0	125 mL per unit	Reference compartment volume	Derived
E_SPEC	13.5 cmH_2O	Specific elastance used for strain	Literature anchor
ε_th	0.30	Linear-strain reference threshold	Literature anchor
σ_th	49.8 kPa	Stress-domain reference threshold	Derived scale
K_E	166 kPa	Stress–strain scale-bridging modulus	Derived
K_LOCAL	6.0 ceiling	Local junction stress concentration bound	Literature bound
η_hyst	0.15	Hysteretic strain-energy fraction	Fixed assumption
τ_ve	0.5	Viscoelastic time constant	Fixed assumption
κ_T	0.075	Rate-dependent transient coefficient	Fixed assumption
γ_surf	0.60	Effect of surfactant state on opening pressure	Direction anchored
Opening width	2 cmH_2O	Width of sigmoid opening transition	Fixed assumption
Refractory boost	1.6	Dependent opening-pressure amplification	Provisional assumption

Table S4. Compartmental mechanics and threshold parameter.

Parameter	Value	Role	Basis
w_ε, w_σ	0.60, 0.40	Relative strain and stress contributions to direct injury	Fixed assumption
p_accel	2.0	Suprathreshold acceleration exponent	Fixed assumption
k_soft	8.0	Softplus sharpness	Fixed assumption
λ	0.05	Direct strain–stress pathway scale	Fixed assumption
κ_F	0.25	Cumulative strain-history susceptibility modifier	Ablated in S8
q_F	2.0	Strain-history accumulation exponent	Fixed assumption
κ_RD	0.03	RACE-mediated atelectrauma scale	Timescale anchor
AMP_RD	6.0	Post-barrier RACE amplification	Sensitivity tested in S6
N_crit	28 to 10 events	Nondependent-to-dependent barrier cycle capacity	Sensitivity tested in S6
REPAIR_RD	0.15	Stable-compartment barrier repair factor	Fixed assumption
SPREAD_RD	0.35	Mean-field regional injury spread	Sensitivity tested in S6
κ_surf up	0.05	Surfactant deterioration under instability	Fixed assumption
κ_surf rec	0.02	Surfactant recovery under stability	Fixed assumption
ψ_floor	0.0006	Irreducible pathway floor	Homeostatic boundary
ρ	0.020	Recovery rate in CDI update	Fixed assumption
λ_mem	0.004	Damage-memory recovery rate	Fixed assumption
δ	2.5	CDI-to-compliance mapping coefficient	Clinical floor anchor
NIEMAN_CAP	0.0155 h^{-1}	Maximum structural-compliance decline rate	Experimental anchor [9]

Table S5. Regional exposure, tissue-state, and recovery parameters.

Parameter	Value	Role	Basis
RR_ref	15 breaths/min	Reference rate for RACE event scaling	Calibrated range
Δt	0.1 h	State-update interval	Numerical design
Horizon	96 h	Canonical experiment duration	Protocol design
VT start	450 mL	Starting volume in Experiments 1, 3, and 4	Clinical order
VT floor	390 mL	Lower safety-rail boundary	Dead-space constraint
Pplat trigger	33 cmH_2O	Tidal-volume reduction trigger	ARDSNet-derived
PEEP candidates	6 to 24 cmH_2O	Controller search range	Protocol design
Controller interval	6 h	PEEP reassessment interval	Protocol design
RACE cutoff	0.05	Operational RACE-active compartment threshold	Sensitivity tested
Stress cutoff	$r\sigma > 1.0$	Suprathreshold compartment count	Sensitivity tested
Aeration floor	0.65	Aggressive comparator openness target	Fixed assumption
Aggressive PEEP	8 start; 22 cap	Aeration-targeted comparator range	Protocol design
Aggressive step	2 cmH_2O per 4 h	Aeration-targeted titration	Protocol design
κ_hypox	0.60	Legacy low-openness objective penalty	Provisional assumption
HEMO_W	0.010	Heuristic high-PEEP penalty above 18; not a hemodynamic model	Provisional assumption

Table S6. Ventilator protocol and controller parameters.

Implementation note. R_BASE = 0.05 remains declared in the archived code for provenance but is unused in the frozen execution path; no reported result depends on it. Respiratory rate affects the RACE event counter relative to RR_ref but does not independently scale the direct strain–stress pathway. The current build should therefore not be used to infer tidal-volume–rate tradeoffs at fixed minute ventilation outside the calibrated range.

S6 One-at-a-Time Model-Parameter Sensitivity

S6.1 Methods

The three provisional parameter groups that define epithelial-barrier resilience and the propagation of RACE-mediated injury were varied one at a time in N = 40 matched virtual lungs generated with seed 20260710. The nondependent and dependent critical RACE counts were scaled together by 0.7 and 1.3; post-barrier RACE amplification was varied from 3.0 to 9.0; and the mean-field regional spread coefficient was varied from 0.20 to 0.50. The phenotype distributions, ventilator protocols, controller logic, 96-h horizon, and all other parameters were unchanged. Four prespecified behaviors were reassessed: severity ordering under practice ventilation, preservation of the early lag, direction of the crossover comparison with PEEP 8, and direction of the crossover comparison with aggressive recruitment.

Setting	Mild %	Severe %	12-h %	PEEP 8 loss %	Aggressive loss %	FAAST loss %	FAAST PEEP	Favors vs PEEP 8 %	Favors vs aggressive %
Frozen baseline	55	38	98	61.4	53.1	41.3	13.7	100	95
N_crit × 0.7	51	36	97	63.6	55.8	43.9	14.0	100	95
N_crit × 1.3	59	39	98	58.5	52.1	39.2	13.5	100	98
AMP_RD 3.0	58	40	98	59.1	52.1	39.7	13.6	100	95
AMP_RD 9.0	54	37	97	62.3	53.6	42.0	13.7	100	95
SPREAD_RD 0.20	58	38	98	58.6	52.6	40.0	13.6	100	95
SPREAD_RD 0.50	53	37	98	63.1	54.3	42.6	13.7	100	95

Table S7. One-at-a-time sensitivity of provisional barrier and RACE-pathway parameters. Mild and severe columns report percentage of structural compliance remaining at 96 h under PEEP 8; other strategy columns report median percentage structural-compliance loss at 96 h.

S6.2 Interpretation

Severity ordering and the early lag were preserved in all seven configurations. The crossover was favorable to PEEP 8 in every matched case under every configuration and was favorable to aggressive recruitment in 95–98% of cases. Median magnitudes moved, as expected, but the prespecified directional behaviors did not reverse. This analysis supports robustness of the central practice comparison within the tested model domain; it does not establish that the provisional parameter values are physiologically calibrated.

S7 Crossover Operational-Threshold Sensitivity

S7.1 Methods

The Experiment 3 independent-seed cohort was regenerated with N = 50 and seed 77777. For each virtual lung, PEEP was swept from 4 to 24 cmH_2O in 1-cmH_2O increments. The crossover selected the lowest PEEP at which the number of suprathreshold compartments equaled or exceeded the number of RACE-active compartments. The same lung was then run for 96 h at PEEP 8 cmH_2O and at the selected crossover PEEP.

Sensitivity analysis varied the RACE cutoff tenfold downward and upward (0.005, 0.05, and 0.5) and varied the stress-ratio cutoff (0.8, 1.0, and 1.2), producing nine operational definitions. The damage engine, cohort, seed, ventilator settings, and safety rail were held fixed. The retrospective model-defined optimum was calculated only as an internal benchmark and is not a clinically available target.

RACE cutoff	Stress cutoff	Median PEEP	Median loss %	Improvement vs PEEP 8	Favors crossover %	Within 2 points of optimum %
0.005	0.8	12.0	41.3	20.7	100	46
0.005	1.0	13.0	40.7	21.3	100	84
0.005	1.2	16.0	40.5	21.5	100	76
0.050	0.8	10.0	46.3	15.7	100	0
0.050	1.0	12.0	42.5	19.6	100	30
0.050	1.2	14.5	39.1	22.9	100	96
0.500	0.8	9.0	52.4	9.6	92	0
0.500	1.0	10.0	46.1	15.9	100	0
0.500	1.2	13.0	40.6	21.4	100	76

Table S8. Operational-threshold sensitivity of the compartmental crossover. Loss is modeled structural-compliance loss at 96 h.

S7.2 Interpretation

Selected PEEP was threshold-dependent: the median ranged from 9 to 16 cmH_2O, and median modeled loss ranged from 39.1% to 52.4%. The directional comparison with PEEP 8 was more stable. Every threshold combination favored the crossover in at least 92% of matched cases, and seven of nine favored it in every matched case. Proximity to the retrospective model-defined optimum was less stable, ranging from 0% to 96%. The analysis therefore supports a narrow conclusion: the crossover moved PEEP away from under-recruitment in this model, but its exact selection and apparent optimality depend on how the regional states are operationally defined.

S8 Strain-History Modifier Ablation

S8.1 Methods

Experiment 1 was rerun with the frozen susceptibility modifier $\kappa_F = 0.25$ and with $\kappa_F = 0$. No other parameter, cohort definition, strategy, safety rail, or simulation horizon was changed. Setting κ_F to zero removes the influence of accumulated suprathreshold strain history on the current direct strain–stress pathway while leaving the underlying strain, stress, RACE, barrier, CDI, recovery, and compliance-update equations intact.

Strategy	Median PEEP κ_F=0.25	Median loss κ_F=0.25	Median PEEP κ_F=0	Median loss κ_F=0
PEEP 5	5.0	68.8	5.0	68.8
PEEP 8	8.0	61.4	8.0	61.4
PEEP 10	10.0	46.5	10.0	46.4
PEEP 15	15.0	39.3	15.0	37.6
Aggressive recruitment	20.2	52.7	20.2	43.7
Legacy summed-dose	18.2	43.2	18.2	38.3
FAAST crossover	13.5	40.7	13.5	39.6

Table S9. Experiment 1 strain-history susceptibility-modifier ablation. Loss is median modeled structural-compliance loss at 96 h.

S8.2 Interpretation

Removing the provisional modifier changed median FAAST loss from 40.7% to 39.6%; FAAST remained favorable to PEEP 8 in 100% of matched cases and to aggressive recruitment in 96.7%. The central comparison with practice therefore did not depend on the modifier. The ablation did alter absolute injury estimates at higher PEEP and narrowed the separation from aggressive recruitment, confirming that the parameter affects magnitude and should not be treated as clinically calibrated.

S9 Reproducibility and Execution Map

The frozen evaluation build, model specification, run instructions, core experiment drivers, original one-at-a-time sensitivity analysis, and seeds are available in Zenodo version 1.0.0 at https://doi.org/10.5281/zenodo.21577653 [15]. Two additional scripts accompanying this revision—jcm_threshold_sensitivity.py and jcm_fatigue_modifier_ablation.py—reproduce the analyses reported in Tables S8 and S9, respectively. The reference implementation is self-contained apart from NumPy.

File	Purpose
ventguard_lung_model.py	Frozen twenty-compartment lung substrate, state updates, and controller logic
run_experiment.py	Controller-blind nine-gate correspondence fence
experiment1_recruitment_and_dose.py	Experiment 1 seven-arm recruitment and strategy comparison
faast_observability.py	Experiment 2 regional observability analysis
peep_optimization.py	Experiment 3 PEEP sweep and crossover analysis
experiment4_combined_effect.py	Experiment 4 matched 2 × 2 factorial with safety rail on and off
sensitivity.py	One-at-a-time sensitivity analysis of provisional constants
jcm_threshold_sensitivity.py	Operational RACE and stress-threshold sensitivity reported in Table S8
jcm_fatigue_modifier_ablation.py	Strain-history modifier ablation reported in Table S9
MODEL_SPEC.md and METHODS.md	Human-readable model and methods specification

Table S10. Public reproducibility files and their role.

Execution details. The canonical horizon was 96 h with a state-update interval of 0.1 h. The correspondence fence, Experiment 1, and Experiment 4 used seed 20260710; the Experiment 3 independent-seed cohort used seed 77777. Experiment 2 used prescribed phenotypes and a 24-h horizon. The archived requirements file specifies NumPy 2.4.4.

Interpretive boundary. Reproducibility demonstrates that the declared equations, parameters, and protocols generate the reported outputs. It does not substitute for external physiological or clinical validation. The present model does not calculate gas exchange, dead space, pH, cardiac output, right-ventricular loading, patient–ventilator interaction, inflammatory kinetics, or phenotype transition. Any future bedside implementation must operate inside a clinically viable envelope and remain subordinate to these measurements and to clinician judgment.

Supplementary References

1. Panwar R, Madotto F, Laffey JG, van Haren FMP; LUNG SAFE Investigators and the ESICM Trials Group. Compliance phenotypes in early acute respiratory distress syndrome before the COVID-19 pandemic. *Am J Respir Crit Care Med*. 2020;202(9):1244-1252. doi:10.1164/rccm.202005-2046OC.
2. Park D, Lee GJ, Kim HY, Ryu JS. Different characteristics of ventilator application between tracheostomy- and noninvasive positive pressure ventilation patients with amyotrophic lateral sclerosis. Medicine (Baltimore). 2017;96(26):e7376. doi:10.1097/MD.0000000000007376.
3. Gattinoni L, Caironi P, Cressoni M, et al. Lung recruitment in patients with the acute respiratory distress syndrome. *N Engl J Med*. 2006;354(17):1775-1786. doi:10.1056/NEJMoa052052.
4. Amato MBP, Meade MO, Slutsky AS, et al. Driving pressure and survival in the acute respiratory distress syndrome. *N Engl J Med*. 2015;372(8):747-755. doi:10.1056/NEJMsa1410639.
5. Caironi P, Langer T, Carlesso E, Protti A, Gattinoni L. Time to generate ventilator-induced lung injury among mammals with healthy lungs: a unifying hypothesis. *Intensive Care Med*. 2011;37(12):1913-1920. doi:10.1007/s00134-011-2388-9.
6. Yamaguchi E, Yao J, Aymond A, Chrisey DB, Nieman GF, Bates JHT, Gaver DP. Electric Cell-Substrate Impedance Sensing (ECIS) as a platform for evaluating barrier-function susceptibility and damage from pulmonary atelectrauma. *Biosensors* (Basel). 2022;12(6):390. doi:10.3390/bios12060390.
7. Gattinoni L, Tonetti T, Quintel M. Regional physiology of ARDS. *Crit Care*. 2017;21(Suppl 3):312. doi:10.1186/s13054-017-1905-9.
8. Acute Respiratory Distress Syndrome Network; Brower RG, Matthay MA, Morris A, Schoenfeld D, Thompson BT, Wheeler A. Ventilation with lower tidal volumes as compared with traditional tidal volumes for acute lung injury and the acute respiratory distress syndrome. *N Engl J Med*. 2000;342(18):1301-1308. doi:10.1056/NEJM200005043421801.
9. Nieman GF, Satalin J, Andrews P, Aiash H, Habashi NM, Gatto LA. Personalizing mechanical ventilation according to physiologic parameters to stabilize alveoli and minimize ventilator induced lung injury (VILI). *Intensive Care Med Exp*. 2017;5(1):8. doi:10.1186/s40635-017-0121-x.
10. Protti A, Andreis DT, Monti M, et al. Lung stress and strain during mechanical ventilation: any difference between statics and dynamics? *Crit Care Med*. 2013;41(4):1046-1055.doi:10.1097/CCM.0b013e31827417a6.
11. Gefen A, Elad D, Shiner RJ. Analysis of stress distribution in the alveolar septa of normal and simulated emphysematic lungs. *J Biomech*. 1999;32(9):891-897. doi:10.1016/s0021-9290(99)00092-8.
12. Sarabia-Vallejos MA, Zúñiga M, Hurtado DE. The role of three-dimensionality and alveolar pressure in the distribution and amplification of alveolar stresses. *Sci Rep*. 2019;9:8783. doi:10.1038/s41598-019-45343-4.Roy S, Sadowitz B, Andrews P, et al. Early stabilizing alveolar ventilation prevents acute respiratory distress syndrome: a novel timing-based ventilatory intervention to avert lung injury. *J Trauma Acute Care Surg*. 2012;73(2):391-400. doi:10.1097/TA.0b013e31825c7a82.
13. Champagne MS. Fatigue-aware alveolar stress and strain tracking: a theoretical biomechanics framework for regional stress-strain dose accumulation in ventilator-induced lung injury. *Front Bioeng Biotechnol*. Manuscript under review.
14. Bellani G, Laffey JG, Pham T, et al; LUNG SAFE Investigators and the ESICM Trials Group. Epidemiology, patterns of care, and mortality for patients with acute respiratory distress syndrome in intensive care units in 50 countries. *JAMA*. 2016;315(8):788-800. doi:10.1001/jama.2016.0291
15. Champagne, M.S. FAAST-2 Regional Lung Intelligence: Frozen Reproducibility Release v1.0.0;Zenodo: Geneva, Switzerland, 2026. https://doi.org/10.5281/zenodo.21577653.
